# A self-reporting AIE probe with a built-in singlet oxygen sensor for targeted photodynamic ablation of cancer cells†
†Electronic supplementary information (ESI) available: Synthesis and characterization of the intermediates; molecular orbital data. See DOI: 10.1039/c5sc03583j


**DOI:** 10.1039/c5sc03583j

**Published:** 2015-11-23

**Authors:** Youyong Yuan, Chong-Jing Zhang, Shidang Xu, Bin Liu

**Affiliations:** a Department of Chemical and Biomolecular Engineering , National University of Singapore , 4 Engineering Drive 4 , 117585 , Singapore; b Institute of Materials Research and Engineering , Agency for Science, Technology and Research (A*STAR) , 3 Research Link , 117602 , Singapore . Email: cheliub@nus.edu.sg

## Abstract

The real-time monitoring of reactive oxygen species (ROS, particularly singlet oxygen) generation during photodynamic therapy is a great challenge due to the extremely short half-life and small radius of action. To tackle this issue, we herein report a bioprobe composed of a red emissive photosensitizer (PS) with aggregation-induced emission (AIE) characteristics and a fluorogenic green emissive rhodol dye conjugated *via* a singlet oxygen cleavable aminoacrylate (AA) linker. The probe emits red fluorescence in water, and the red emissive PS can be used for probe self-tracking. Upon image-guided light irradiation, the generated singlet oxygen cleaves the AA linker to yield green fluorescence turn-on of rhodol, which offers real-time and *in situ* monitoring of singlet oxygen generation during photodynamic ablation of cancer cells, providing a strategy for the early evaluation of the therapeutic effect.

## Abstract 2

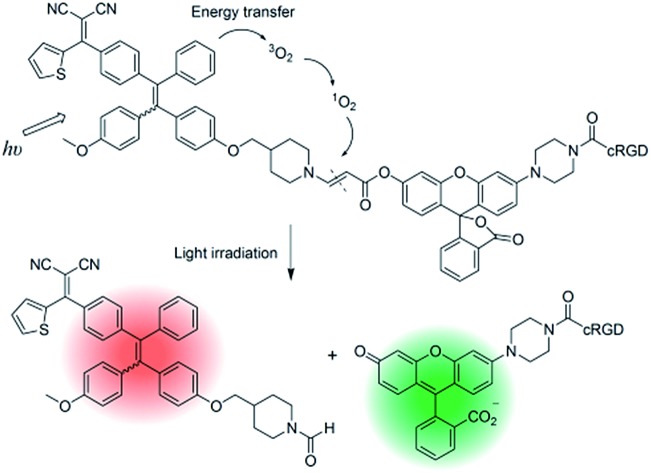
A probe for the *in situ* monitoring of singlet oxygen generation during targeted theranostic photodynamic therapy is developed based on a photosensitizer with aggregation-induced emission (AIE) characteristics and conjugated to a fluorogenic rhodol dye *via* a singlet oxygen cleavable linker.

## Introduction

Targeted drug delivery has been extensively studied for cancer therapy.^1^ However, precisely answering when, where, and how the therapeutic agents are delivered and what their functions are remain challenging. Recently, theranostic delivery systems that combine therapy and diagnostic imaging have attracted great attention in biomedical research.^2^ They offer the opportunity to evaluate therapeutic regimes and provide useful information for dose adjustment and prognosis of individual patients to achieve the ultimate goal of personalized medicine.^3^ The systems require real-time monitoring of cancer therapy, particularly if the process can be detected in a non-invasive manner. Recently, a fluorophore or contrast agent conjugated with a chemotherapy drug *via* a tumor-specific responsive linker has been developed for the real-time and *in situ* reporting of drug activation.^4^ The design principle mainly relies on the change of fluorescence intensity or magnetic resonance imaging (MRI) signal concomitantly occurring with the drug activation.

Compared to chemotherapy drugs, photosensitizer (PS) drugs have recently received increased attention because photodynamic therapy (PDT) does not acquire drug resistance, and it shows limited side effects as its toxicity is light-controllable.^5^ PDT is based on the concept that PSs generate cytotoxic reactive oxygen species (ROS), particularly singlet oxygen, upon light irradiation to induce cell death. Although some fluorescent probes have been developed for singlet oxygen detection based on fluorescence changes,^6^ the separate administration of the PS and the probe does not guarantee them to have the same location in cells.6*b* As singlet oxygen has very short lifetime (<40 ns) and a small radius of action (<20 nm),^7^ it remains a challenge to monitor singlet oxygen generation during PDT in real-time and *in situ* at the subcellular level. The design of a probe that can simultaneously image and ablate cancer cells with real-time monitoring of the singlet oxygen generation during PDT is technically challenging and it is practically not available yet.

Traditional PSs, such as porphyrin, with large planar structures show aggregation-caused quenching (ACQ) with weak fluorescence and reduced singlet oxygen generation in solid state or in aggregates due to π–π stacking.^8^ Recently, fluorogens with aggregation-induced emission characteristics (AIEgens) have received extensive attention for biosensors and bioimaging.^9^ Distinctively, AIEgens emit intensively in aggregated state due to the restriction of intramolecular motions.9*a* More recently, AIEgen PSs with high signal-to-background ratio for fluorescence imaging and efficient singlet oxygen generation in aggregates have been developed for image-guided PDT.^10^

As a proof of concept, in this contribution, by taking advantages of AIE PSs, we designed a probe for imaging, ablating cancer cells and real-time monitoring of singlet oxygen generation during PDT. The probe is composed of an AIE PS and a fluorogenic rhodol dye conjugated through a singlet oxygen cleavable aminoacrylate (AA) linker.^11^ The probe is red emissive in aqueous media, which can be utilized for probe self-tracking. Upon image-guided light irradiation, the green fluorescence of rhodol intensifies greatly as the generated singlet oxygen can cleave the AA linker to release the highly emissive fluorophore 6-hydroxy-3*H*-xanthen-3-one,^12^ which can be used for the real-time and *in situ* monitoring of singlet oxygen generation during PDT.

## Results and discussion

**Scheme 1:**
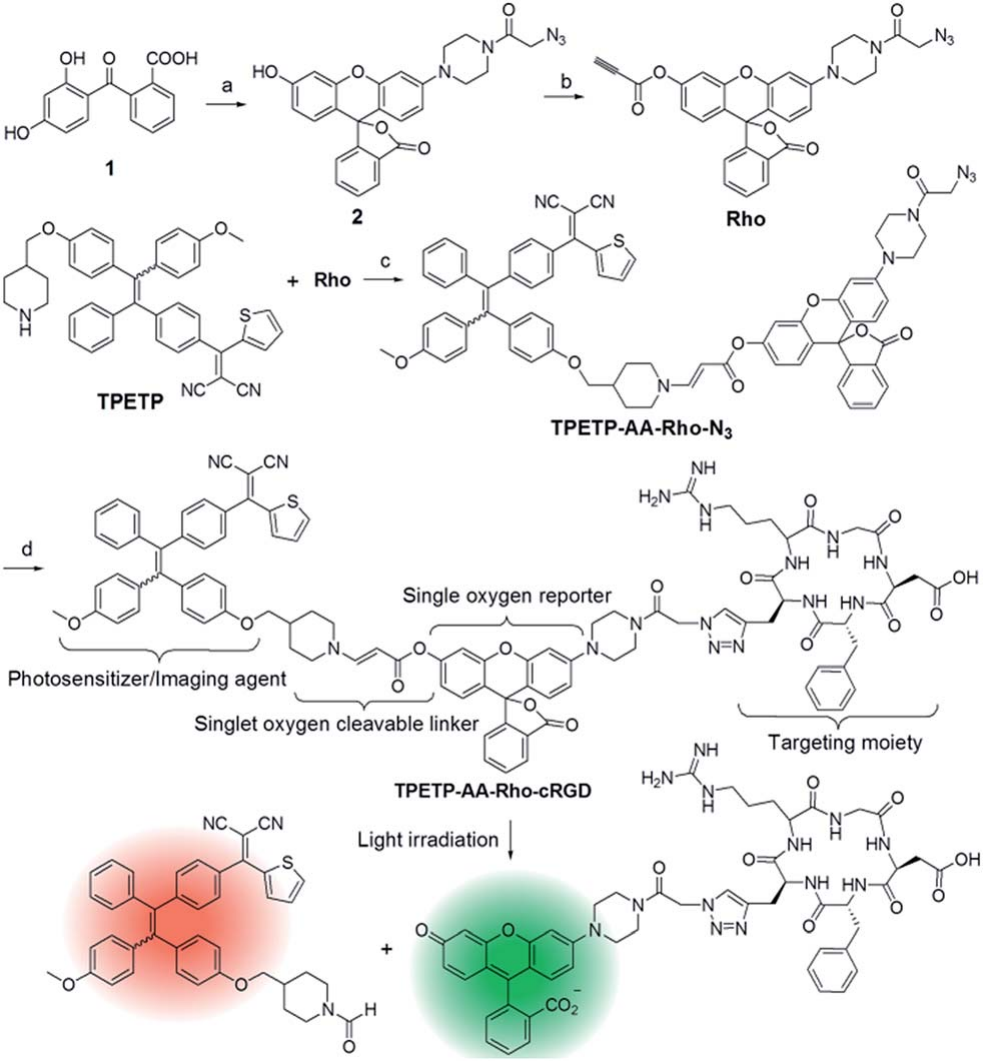
(A) Synthesis of the theranostic probe TPETP-AA-Rho-cRGD and a schematic representation of the proposed singlet oxygen self-reporting mechanism. Reagents: (a) 1-(3-hydroxyphenyl)-piperazine, TFA; then 2-azidoacetic acid, DMAP, EDC, DMF; (b) propiolic acid, DMAP, DCC, DCM; (c) TEA, THF; (d) cRGD-alkyne, CuSO_4_, sodium ascorbate, DMSO and water. TFA: trifluoroacetic acid; EDC: *N*-(3-dimethylaminopropyl)-*N*′-ethylcarbodiimide hydrochloride; TEA: triethylamine; DMF: dimethylformamide; DCM: dichloromethane; DMAP: 4-(dimethylamino)pyridine; DCC: *N*,*N*′-dicyclohexylcarbodiimide; THF: tetrahydrofuran.

The synthetic route to the probe is shown in Scheme 1. Compound **2** was obtained by the reaction of compound **1** with 2-azidoacetic acid and then it was further esterified with propiolic acid. The AIE PS (TPETP) was synthesized according to Scheme S1.† It reacted with compound **3** through an yne-amine click reaction to yield TPETP-AA-Rho-N_3_. The probe was obtained after the click reaction between TPETP-AA-Rho-N_3_ and alkyne-functionalized cRGD, and was denoted as TPETP-AA-Rho-cRGD. The detailed synthetic procedure and characterization data of the probe are shown in Fig. S1–S8.†

**Fig. 1:**
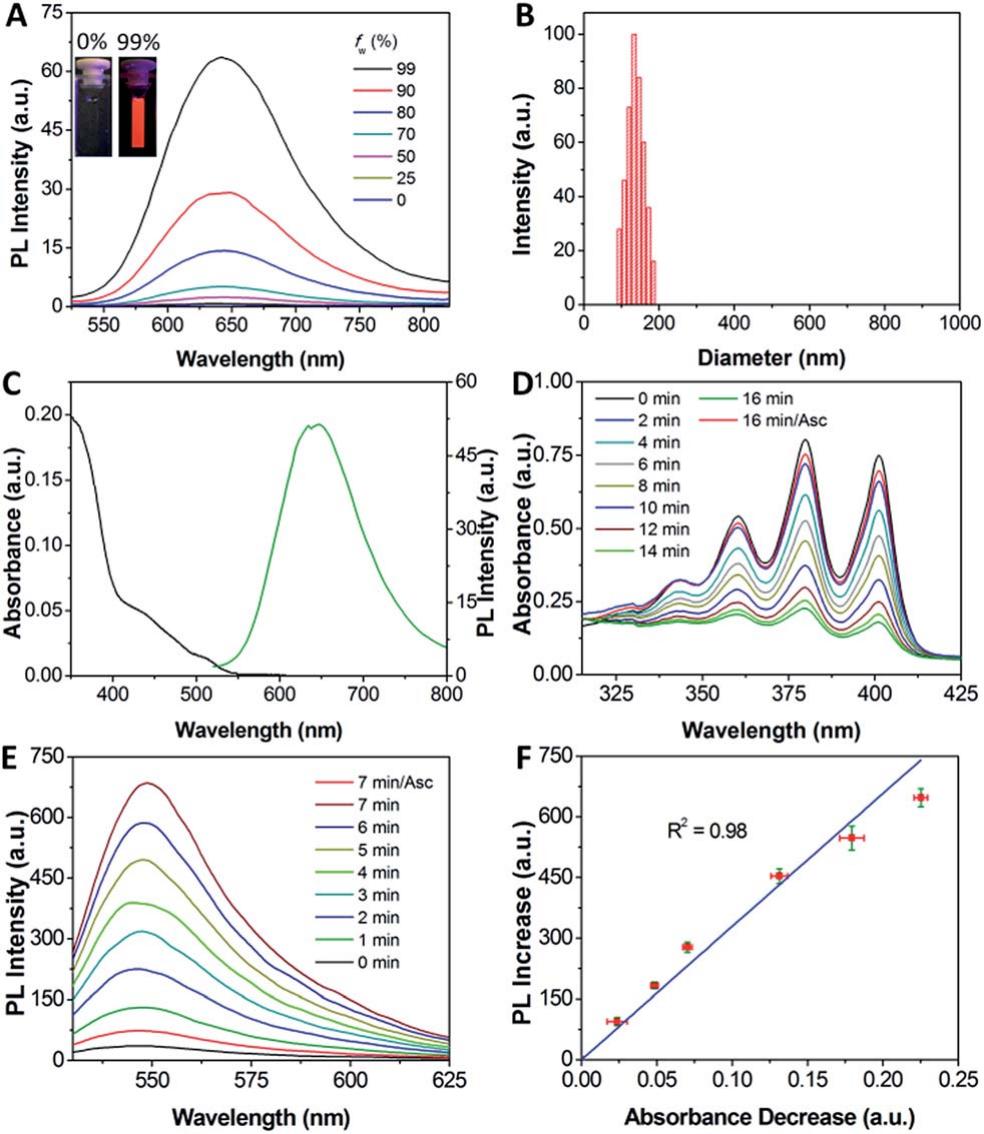
(A) Photoluminescence (PL) spectra of TPETP (10 μM) in DMSO/water mixtures with different fractions of water (*f*_w_). (B) Size distribution of TPETP-AA-Rho-cRGD (10 μM) in DMSO/PBS (v/v = 1/199). (C) UV-vis absorption and emission spectra of the probe (10 μM) excited at 430 nm. (D) Time dependent absorption changes of 9,10-anthracenediyl-bis(methylene)dimalonic acid (ABDA) mixed with the probe (10 μM) upon visible light (*λ* = 400–700 nm) irradiation or in the presence of singlet oxygen scavenger ascorbic acid (Asc). The power density was 0.10 W cm^–2^. (E) Time dependent PL intensity change of the probe (10 μM) with light irradiation (*λ*_ex_: 510 nm). (F) Correlation between the absorption decrease at 400 nm in (D) and the PL intensity increase at 550 nm in (E).

The absorption spectrum of TPETP is shown in Fig. S9A,† which shows a shoulder peak at 430 nm and an emission maximum at 650 nm. The quantum yield (*Φ*) of TPETP is determined to be 0.13 ± 0.01 using 4-(dicyanomethylene)-2-methyl-6-(4-dimethylaminostyryl)-4*H*-pyran (DCM) as the standard (*Φ* = 0.43). The AIE property of TPETP was confirmed by studying its PL spectra in DMSO/water mixtures with different water fractions (Fig. 1A). The formation of TPETP aggregates in aqueous solution was also confirmed (Fig. S9†). The probe TPETP-AA-Rho-cRGD forms aggregates in DMSO and phosphate buffered saline (PBS) mixture (v/v = 1/199) with sizes of 140 ± 13 nm (Fig. 1B). The absorption and emission spectra of the probe are shown in Fig. 1C. The probe shows an absorption peak at 430 nm with a very weak shoulder at 510 nm, indicating that Rho exists in the ring closed form.^12^ Upon excitation at 430 nm, the emission spectrum of the probe is similar to that of TPETP and the green fluorescence of Rho is weak. The singlet oxygen generation of the probe upon visible light (*λ* = 400–700 nm) irradiation was studied using 9,10-anthracenediyl-bis(methylene)dimalonic acid (ABDA) as an indicator. As shown in Fig. 1D and S10,† only in the presence of TPETP was a decrease in the ABDA absorption observed, an indication of singlet oxygen generation. The singlet oxygen quantum yield (*Φ*) of TPETP in DMSO/PBS (v/v = 1/199) was determined to be 0.68 using Rose Bengal (RB) as the standard photosensitizer (*Φ*_RB_ = 0.75 in water), which is much higher than the clinically used PSs such as Photofrin® (*Φ* = 0.28) or Laserphyrin® (*Φ* = 0.48).^7^ The absorbance decrease of ABDA was inhibited in the presence of the singlet oxygen scavenger ascorbic acid (Asc).

The photoluminescence (PL) change of the probe upon visible light irradiation was also monitored over different durations of light irradiation. As shown in Fig. 1E, the fluorescence signal of Rho shows a quick and steady increase upon light irradiation, and the fluorescence is 19-fold brighter than the original Rho emission from the probe after light irradiation for 7 minutes. In contrast, no significant fluorescence increase is observed when Asc is present. It should be noted that the absorption and emission of the compounds Rho and TPETP do not change upon light irradiation under the same conditions. It was also found that the probe fluorescence is pH independent, but after light illumination the fluorescence of the green emissive product is pH dependent (Fig. S11†), which agrees with the fluorescent properties of 6-hydroxy-3*H*-xanthen-3-one.^12^ The selectivity of the probe towards different ROS including singlet oxygen (^1^O_2_), hydrogen peroxide (H_2_O_2_), peroxynitrite (ONOO^–^), superoxide (O_2_˙^–^), and hydroxyl radicals (˙OH) in N_2_-purged PBS was also studied. As shown in Fig. S11C,† the fluorescence of Rho shows significant enhancement only in the presence of singlet oxygen, which confirms that the AA linker can be selectively cleaved by singlet oxygen.11*b* Reverse-phase HPLC and mass analyses further confirmed the anticipated linker cleavage after light irradiation (Fig. S12†). To assess whether the probe can monitor the singlet oxygen generation *in situ*, we evaluated the correlation between the absorbance decrease of ABDA and the fluorescence increase of Rho. The results shown in Fig. 1F clearly demonstrate that the fluorescence increase of Rho is linearly (*R*^2^ = 0.98) correlated with singlet oxygen generation, which indicates that the fluorescence changes of Rho can be used for real-time monitoring of singlet oxygen generation during PDT.

**Fig. 2:**
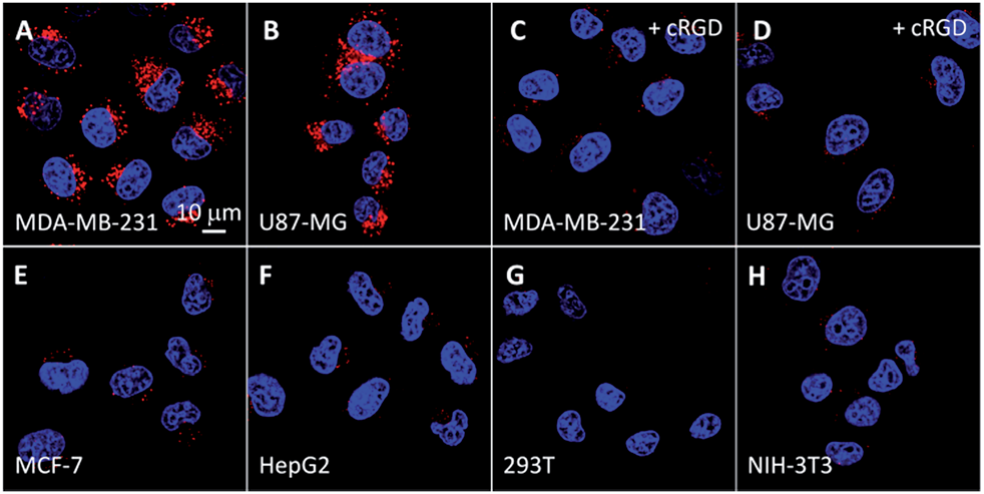
(A–H) Confocal images of the probe (10 μM) incubated with different cell lines for 4 h. For C and D, the cells were pretreated with free cRGD (100 μM). Red fluorescence (TPETP, *E*_x_: 405 nm; *E*_m_: >650 nm); blue fluorescence (Hoechst, *E*_x_: 405 nm; *E*_m_: 430–470 nm).

Receptor mediated cancer therapy is attractive as it can facilitate the cellular internalization through receptor mediated endocytosis. To demonstrate the feasibility of cancer targeting, the probe was incubated with α_v_β_3_ integrin overexpressed MDA-MB-231 and U87-MG cancer cells and low α_v_β_3_ integrin expressed MCF-7 and HepG2 cancer cells as well as NIH/3T3 and 293T normal cells. The red fluorescence in U87-MG and MDA-MB-231 cells increases with incubation time (Fig. S13 and S14†) and the intensities are much higher than the other cell lines (Fig. 2). However, the fluorescence signal is greatly inhibited when the cells are pretreated with cRGD, confirming that the probe is internalized by cancer cells *via* receptor-mediated endocytosis. To identify the cellular location of the probe after α_v_β_3_ integrin mediated endocytosis, colocalization experiments were performed by co-staining the probe labelled cells with a fluorescent endo-/lysosome tracker or mitochondria tracker. The co-localized results shown in Fig. S13† demonstrate that the probe is entrapped in endo-/lysosomes, but not mitochondria, after the cellular internalization, which agrees with the nanoaggregate nature of the probe.

**Fig. 3:**
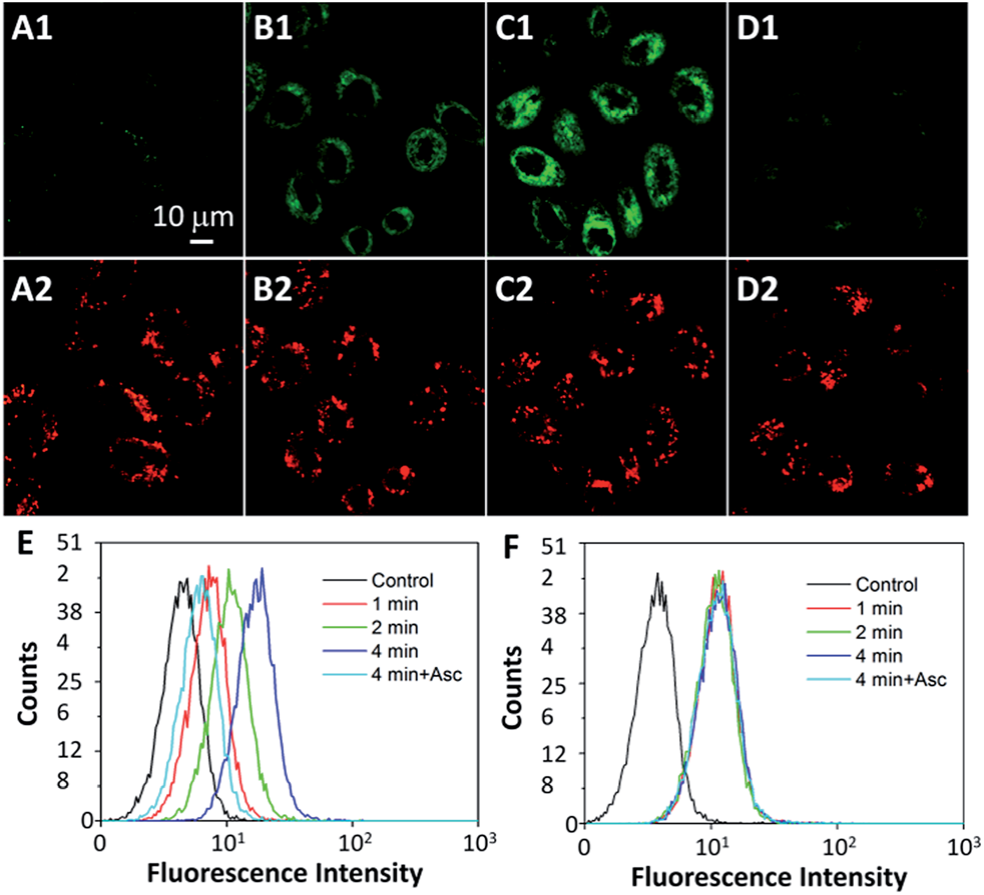
CLSM images of the probe (10 μM) incubated MDA-MB-231 cells with light irradiation at a power density of 0.10 W cm^–2^ for (A) 0 min, (B) 2 min, (C) 4 min and (D) 4 min in the presence of Asc (100 μM). (A1–D1) Green fluorescence (Rho, *E*_x_: 488 nm, *E*_m_: 505–525 nm); (A2–D2) red fluorescence (TPETP, *E*_x_: 405 nm; *E*_m_: >650 nm). (E, F) Flow cytometric analysis of Rho (E) and TPETP (F) fluorescence after light irradiation.

The intracellular singlet oxygen generation induced Rho fluorescence change was studied by confocal microscopy. As shown in Fig. 3, the fluorescence signal is weak without light irradiation, but the fluorescence of Rho is increased along with the light irradiation while that of TPETP remains constant. After 4 min of light irradiation, the green fluorescence is very strong. In contrast, the fluorescence of Rho remains weak after light irradiation when the singlet oxygen scavenger Asc is presented. The fluorescence changes of Rho and TPETP upon light irradiation were also confirmed by flow cytometric analysis (Fig. 3E and F). Similar phenomena were also observed in U87-MG cells (Fig. S15†). The real-time and *in situ* monitoring of the Rho fluorescence change provides a convenient and reliable strategy for self-feedback of singlet oxygen generation during PDT.

**Fig. 4:**
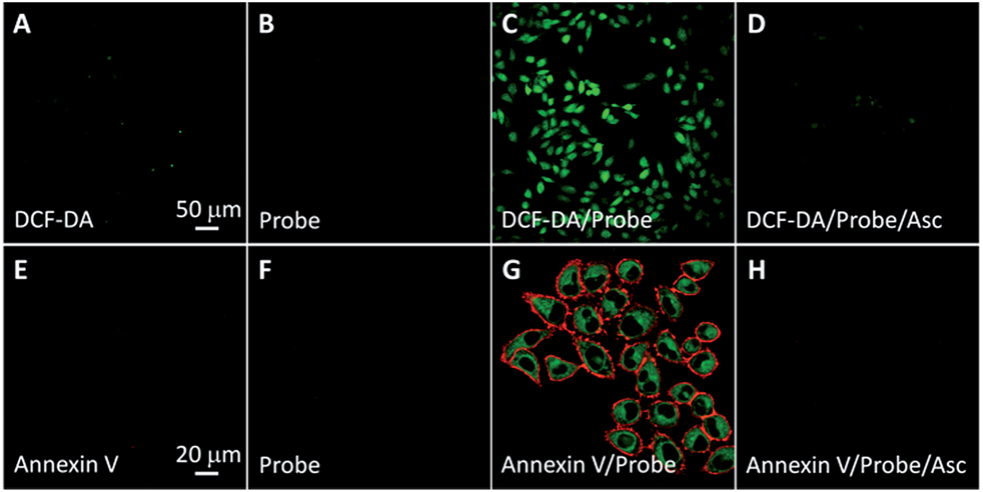
(A–D) CLSM images showing the ROS generation of TPETP in MDA-MB-231 cells after different treatments using 2′,7′-dichlorofluorescin diacetate (DCF-DA, 2 μM) as an indicator. The green fluorescence is from DCF-DA (*E*_x_: 488 nm, *E*_m_: 505–525 nm). (A–D) share the same scale bar of 50 μm. (E–H) Cell apoptosis imaging after different treatments with light irradiation using annexin V-Cy5 (1 μM) as indicator. The power density was 0.10 W cm^–2^. Green fluorescence (Rho, *E*_x_: 488 nm; *E*_m_: 505–525 nm); red fluorescence (Cy5, *E*_x_: 633 nm, *E*_m_: >650 nm). (E–H) share the same scale bar of 20 μm.

The singlet oxygen generation of TPETP in cells upon light irradiation was studied using 2′,7′-dichlorofluorescin diacetate (DCF-DA) as a cell-permeable indicator. DCF-DA is non-fluorescent but can be oxidized by ROS to yield highly fluorescent 2′,7′-dichlorofluorescein (DCF). As shown in Fig. 4A–D, strong green fluorescence can only be observed when the cells were treated with TPETP followed by light irradiation. When the singlet oxygen scavenger Asc is added, the DCF fluorescence is negligible which further confirms the singlet oxygen generation. The singlet oxygen induced cell apoptosis was studied by Cy5-tagged annexin V as an indicator. Cy5-annexin V can distinguish apoptotic cells from viable ones as annexin V can bind to phosphatidylserine expressed on apoptotic cellular membrane. As shown in Fig. 4E–H and S16,† the red fluorescence of Cy5 is only observed on cell surfaces when the cells were treated with the probe followed by light irradiation. These results demonstrate that the probe can generate singlet oxygen in cells and induce cell apoptosis after exposure to light.

**Fig. 5:**
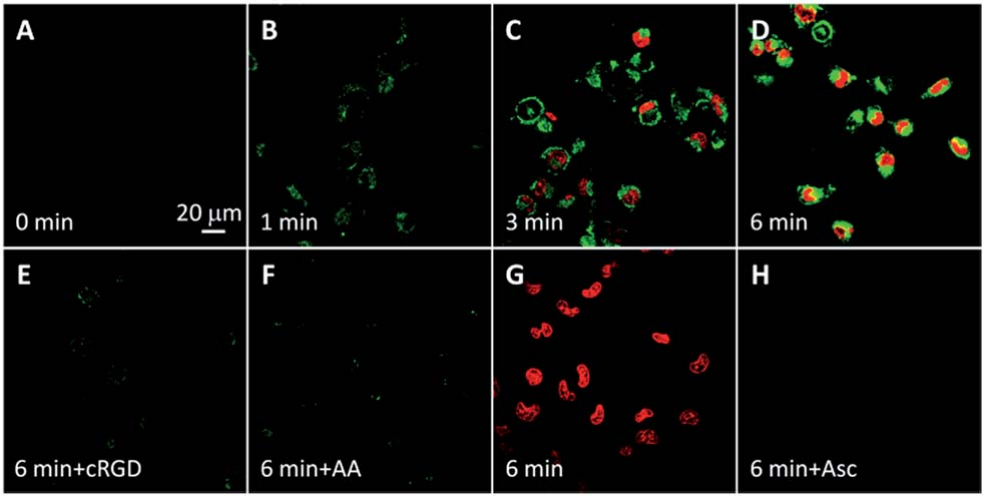
CLSM images of the cell death induced by the probe (A–F, 10 μM) or TPETP (G, H, 10 μM) incubated MDA-MB-231 cells with light irradiation at a power density of 0.10 W cm^–2^ for different time durations and further incubated for 4 h before staining with propidium iodide (PI, 2 μM). Green fluorescence (Rho, *E*_x_: 488 nm; *E*_m_: 505–525 nm); red fluorescence (PI, *E*_x_: 543 nm, *E*_m_: 610–640 nm). Due to the low absorbance of TPETP at 543 nm, its spectral overlap with PI is negligible.

The probe-induced cell necrosis upon light irradiation was subsequently monitored by propidium iodide (PI) as an indicator, which only stains the nuclei of necrotic cells. The probe incubated MDA-MB-231 and U87-MG cells were irradiated with light for different time with TPETP as positive control. As shown in Fig. 5 and S17,† before light irradiation, both green fluorescence of Rho and red fluorescence of PI are negligible. Along with the light irradiation, the green fluorescence is increased and the red fluorescence from the nuclei is observed in some cells, indicating the generated singlet oxygen can turn on the Rho fluorescence and induce cell necrosis. The fluorescence signals of Rho and PI are weak when the cells were pretreated with cRGD or in the presence of Asc. To confirm that the cell necrosis is induced by the singlet oxygen generation of TPETP with light irradiation, the MDA-MB-231 cells were also treated with free TPETP and strong PI fluorescence can be observed while the fluorescence signal is negligible in the presence of Asc. These results confirm that the fluorescence change of Rho can be used to report singlet oxygen generation and predict the therapeutic effect in real-time.

**Fig. 6:**
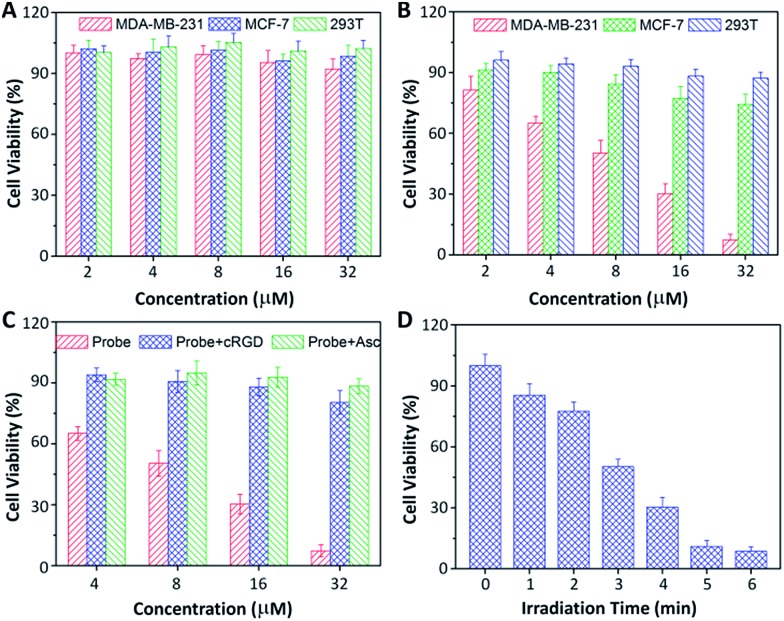
Cytotoxicity of the probe-incubated cells (A) in dark conditions or (B) with light irradiation (4 min, 0.10 W cm^–2^). Cytotoxicity of the probe-incubated MDA-MB-231 cells (C) upon pretreatment with 100 μM of cRGD or Asc or without any pretreatment with light irradiation for 3 min or (D) with different time durations of light irradiation.

Low dark toxicity but high toxicity upon light irradiation is highly essential for phototherapy to minimize side effects and enhance the therapeutic outcome. The cytotoxicity of the probe-incubated cells in the dark or with light irradiation was evaluated by MTT assay. As shown in Fig. 6, the probe shows no apparent toxicity to MDA-MB-231, MCF-7 and 293T cells in dark conditions. Upon light irradiation, the probe only shows significant cytotoxicity to MDA-MB-231 cells while the toxicity to MCF-7 and 293T cells is minimal. The half-maximal inhibitory concentration (IC_50_) of the probe to MDA-MB-231 cells in the dark and upon light irradiation are 219.1 and 8.3 μM, respectively. The phototoxicity index,^13^ which is the ratio between the IC_50_ values in the dark and upon light irradiation, was calculated to be 26.4, indicating that the probe is a good phototherapeutic agent. When the MDA-MB-231 cells were pretreated with free cRGD or Asc, the toxicity of the probe was greatly diminished. In addition, the cytotoxicity of the probe to MDA-MB-231 cells is dependent on the irradiation time. Similar results were also observed for U87-MG, HepG2 and NIH/3T3 cells (Fig. S18†). It should be noted that Rho is almost non-toxic to all the tested cells regardless of light irradiation (Fig. S19†).

## Conclusions

In conclusion, we have developed a novel probe for the theranostic photodynamic ablation of cancer cells. The probe contains an AIE PS which can efficiently generate singlet oxygen upon light irradiation and the intrinsic red fluorescence can be used for image-guided PDT. The cell experiments confirmed that the probe can be selectively taken up by α_v_β_3_ integrin overexpressed cancer cells and localized in endo-lysosomes, which can be tracked by the red fluorescence of TPETP. Upon image-guided light irradiation, the singlet oxygen generation in cells is able to induce cell death, which can be monitored by the fluorescence change of Rho. Therefore, our probe design provides a new strategy for potential theranostic PDT with real-time monitoring of singlet oxygen generation through non-invasive fluorescence signal changes, which offers a new avenue for early evaluation of the PDT therapeutic effect. Further development of the AIE PSs with long wavelength absorption will allow us to conduct *in vivo* PDT in the near future.

## Supplementary Material

Supplementary information
