# Supplementary material for: A self-reporting AIE probe with a built-in singlet oxygen sensor for targeted photodynamic ablation of cancer cells
Source: Chem Sci. 2015 Nov 23;7(3):1862–6. doi: 10.1039/c5sc03583j (PMC5965248; doi:10.1039/c5sc03583j)
Supplement: Supplementary file 1 [file SC-007-C5SC03583J-s001.pdf]

Electronic Supplementary Material (ESI) for Chemical Science.
This journal is © The Royal Society of Chemistry 2015

Supporting Information

A Self-Reporting AIE Probe with a Built-In Singlet Oxygen Sensor for Targeted Photodynamic Ablation of Cancer Cells

Youyong Yuan,[a] Chong-Jing Zhang,[a] Shidang Xu[a] and Bin Liu[*,a,b]

[a]Department of Chemical and Biomolecular Engineering, 4 Engineering Drive 4, National University of Singapore, Singapore, 117585

[b]Institute of Materials Research and Engineering, Agency for Science, Technology and Research (A*STAR), 3 Research Link, Singapore, 117602

Materials and Methods

Materials and characterization: Trifluoroacetic acid (TFA), *N*-(3-dimethylaminopropyl)-*N′*-ethylcarbodiimide hydrochloride (EDC), propiolic acid, *N*, *N′*-dicyclohexylcarbodiimide (DCC), 4-(dimethylamino)pyridine (DMAP), copper(II) sulfate ($CuSO_4$), sodium ascorbate, 9,10-anthracenediyl-bis(methylene)dimalonic acid (ABDA), ascorbic acid (Asc), *N*, *N*-diisopropylethylamine (DIPEA), anhydrous dimethyl sulfoxide (DMSO), anhydrous *N*, *N*-dimethylformanide (DMF), 3-(4,5-dimethylthiazol-2-yl)-2,5-diphenyltetrazolium bromide (MTT), 2′,7′-dichlorofluorescin diacetate (DCF-DA) and other chemicals were all purchased from Sigma-Aldrich or Alfa Aesar and used as received without further purification. Tetrahydrofuran (THF) and dichloromethane were dried by distillation using sodium as drying agent and benzophenone as indicator. All non-aqueous reactions were carried out under nitrogen atmosphere in oven-dried glassware. Deuterated solvents with tetramethylsilane (TMS) as internal reference were purchased from Cambridge Isotope Laboratories Inc.. Alkyne-functionalized cRGD (cyclic(Arg-Gly-Asp-D-Phe)) was customized from GL Biochem Ltd.

Dulbecco's Modified Essential Medium (DMEM) is a commercial product of Invitrogen. Milli-Q water was supplied by Milli-Q Plus System (Millipore Corporation, United States). Phosphate-buffer saline (PBS, 10 ×) buffer with pH = 7.4 is a commercial product of 1st BASE (Singapore). Milli-Q water (18.2 MΩ) was used to prepare the buffer solutions from 10 × PBS stock buffer. 1 × PBS contains NaCl (137 mM), KCl (2.7 mM), Na_2HPO_4 (10 mM), and KH_2PO_4 (1.8 mM). LysoTracker® Red DND-99, MitoTracker® Red FM, propidium iodide (PI), Hoechst 33342, Annexin V-Cy5, fetal bovine serum (FBS) and trypsin-EDTA solution were purchased from Life Technologies.

Visible light (400-700 nm) generated from Cold Light L-150A at a power density of 0.10 W cm^{-2} was used as the irradiation source. The intensities of incident beams were checked by a power and energy meter. NMR spectra were measured on a Bruker ARX 300/400/500 NMR spectrometer. Chemical shifts are reported in parts per million referenced with respect to residual solvent ($CDCl_3$ = 7.26 ppm and $(CD_3)_2SO$ = 2.50 ppm) for ^1H NMR and ($CDCl_3$ = 77.0 ppm and $(CD_3)_2SO$ = 40.0 ppm) for ^{13}C NMR. The extent of reaction was monitored by thin layer chromatography (TLC) using Merck 60 F254 pre-coated silica gel plates with fluorescent indicator UV254. After the plates were subjected to elution in the TLC chamber, the spots were visualized under UV light or using the appropriate stain (I_2, $KMnO_4$, ninhydrin or ceric ammonium molybdate (CAM)). Flash column chromatography was carried out using Merck silica gel (0.040-0.063). A 0.1% trifluoroacetic acid solution in H_2O and acetonitrile was used as the eluent for high-performance liquid chromatography (HPLC) experiments (Agilent). Mass spectra were recorded on Agilent 5975 DIP-MS for electron impact (EI) and the AmaZon X LC-MS for electrospray

ionization (ESI). Particle size and size distribution were determined by laser light scattering (LLS) with a particle size analyzer (90 Plus, Brookhaven Instruments Co., United States) at a fixed angle of 90° at room temperature. TEM images were obtained from a JEOL JEM-2010 transmission electron microscope with an accelerating voltage of 200 kV. UV-vis absorption spectra were taken on a Shimadzu Model UV-1700 spectrometer. Photoluminescence (PL) spectra were measured on a Perkin-Elmer LS 55 spectrofluorometer. All UV and PL spectra were collected at 24 ± 1 °C.

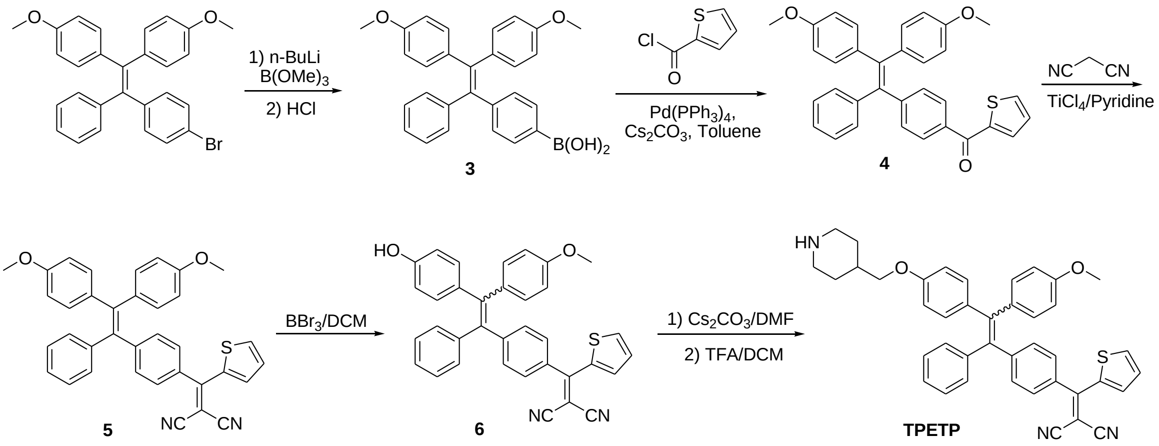

Scheme S1. Synthetic route to TPETP.

Synthesis of compound 1.

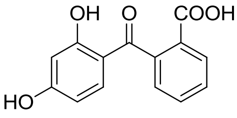

(1)

A solution of fluorescein (6.6 g, 20 mmol) and KOH (22.4 g, 400 mmol) in water (30 mL) was heated at reflux for 5 h. Then the reaction mixture was cooled to 0 °C under ice water bath, acidified with concentrated HCl to pH = 2, and extracted with ether (100 mL×3). The ether extract was concentrated to dryness under reduced pressure. The desired residue was crystallized from CH_2Cl_2 (50 mL) to give compound **1** as a brown solid (4.2 g, 81.4% yield). ^{1}H NMR (400 MHz, DMSO-d_6), δ 13.16 (brs, 1H), 12.23 (s, 1H), 10.70 (s, 1H), 8.00 (dd, J_1 = 1.2 Hz, J_2 = 8.0 Hz, 1H), 7.73 (dt, J_1 = 1.2 Hz, J_2 = 7.6 Hz, 1H), 7.65 (dt, J_1 = 1.2 Hz, J_2 = 7.6 Hz, 1H), 7.42 (dd, J_1 = 1.2 Hz, J_2 = 7.6 Hz, 1H), 6.92 (d, J = 8.8 Hz, 1H), 6.32 (d, J = 2.0 Hz, 1H), 6.29 (dd, J_1 = 2.0 Hz, J_2 = 8.8 Hz, 1H).

Synthesis of compound 2.

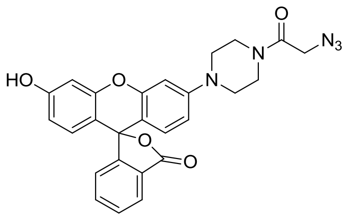

(**2**)

To a pressure tube was added compound **1** (1.24 g, 4.8 mmol), 1-(3-hydroxyphenyl)-piperazine (853 mg, 4.8 mmol) and TFA (20 mL). The reaction mixture was then heated at 95 ^{o}C for 3 h. After cooling down to room temperature, the reaction mixture was poured into ether (300 mL). The resulting precipitate was centrifuged, collected and dissolved in anhydrous DMF (20 mL). To this solution was added 2-azidoacetic acid (410 mg, 4.06 mmol) and DMAP (488 mg, 4.0 mmol). Then EDC (790 mg, 4.11 mmol) in DMF (5 mL) was added. The reaction mixture was stirred at toom temperature for 12 h. The solvent was removed under reduced pressure and the resulting residue was purified with chromatography (hexane: ethyl acetate = 1:1) to give compond **2** as red solid (290 mg, 12.5% yield). ^{1}H NMR (400 MHz, DMSO-d_6), δ 10.10 (s, 1H), 8.00 (m, 1H), 7.80 (dt, J_1 = 1.2 Hz, J_2 = 7.6 Hz, 1H), 7.73 (dt, J_1 = 1.2 Hz, J_2 = 7.6 Hz, 1H), 7.26 (d, J = 7.6 Hz, 1H), 6.82 (d, J = 2.4 Hz, 1H), 6.75 (dd, J_1 = 2.4 Hz, J_2 = 9.2 Hz, 1H), 7.26 (t, J = 2.4 Hz, 1H), 6.55 (m, 3H), 4.19 (s, 2H), 3.60 (m, 2H), 3.45 (m, 2H), 3.31 (m, 4H). HRMS (ESI), calcd for $[M+H]^+$:484.1615, found: 484.1613.

Synthesis of Rho.

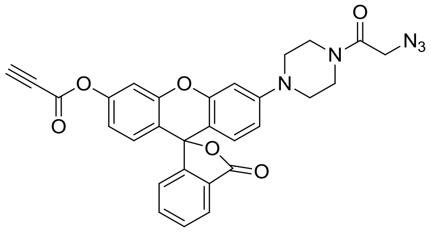

(Rho)

To the solution of compound **2** (50 mg, 0.10 mmol) in DCM (10 mL) was added propiolic acid (21 mg, 0.3 mmol), DMAP (12 mg, 0.1 mmol) and DCC (22 mg, 0.1 mmol). The reacition mixture was stirred at room temperature for 1 h. Then the solvent was removed and the resulting mixture was purified with chromatography (hexane: ethyl acetate = 2:1) to give Rho as a white solid (10 mg, 18.6% yiled). ^{1}H NMR (400 MHz, $CDCl_3$) δ 8.03 (d, J = 7.2 Hz, 1H), 7.68 (m, 2H), 7.26 (m, 2H), 6.85 (m, 1H), 6.72 (m, 2H), 6.63 (m, 1H), 3.98 (s, 2H), 3.79 (m, 2H), 3.55 (m, 2H), 3.28 (m, 4H), 3.12 (s, 1H); MS (ESI), calcd for $[M+H]^+$:536.16, found: 536.60.

Synthesis of compound 3.

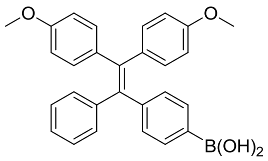

(**3**)

To the solution of compound 1-(2,2-bis(4-methoxyphenyl)-1-phenylvinyl)-4-bromobenzene[1] (7.7 g, 16.3 mmol) in THF (150 mL) was added n-butyllithium (1.6 M in hexane, 16.0 mL) at -78 °C. The mixture was stirred at the same temperature for 2 h. Then trimethyl borate (3.8 mL, 33.4 mmol) was added. The reaction mixture was allowed to warm up and stirred at room temperature for 3 h. The reaction was quenched by addition of HCl solution (3 M, 45 mL) and the resulting solution was stirred at room temperature for 5 h. Then the mixture was diluted with ethyl acetate (100 mL) and brine (200 mL). The organic phase was separated, washed with brine (100 mL × 2), and dried over $MgSO_4$. The mixture was filtered and the filtrate was concentrated under reduced pressure. The residue was subjected to flash chromatography (hexane/ethyl acetate = 10/1 to 2/1) to yield compound **3** as a white solid (2.9 g, 40.8% yield), which was used directly in the next step without further purification.

Synthesis of compound 4.

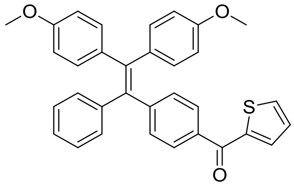

(**4**)

To the suspension of compound **3** (2.9 g, 6.5 mmol) in toluene (80 mL) was added anhydrous cesium carbonate (5.3 g, 16.2 mmol) and tetrakis(triphenylphosphine) palladium(0) (228 mg, 0.32 mmol). Thiophene-2-carbonyl chloride (2.0 g, 13.6 mmol) was added to the above mixture. Then the mixture was stirred at 100 °C for 12 h before it was cooled down to room temperature. The mixture was washed with water (50 mL) and brine (50 mL). The organic layer was dried over $MgSO_4$, filtered and the filtrate was concentrated and purified by chromatography (hexane/ethyl acetate = 50/1 to 10/1) to give compound **4** as an orange solid (2.8 g, 85.8% yield). ^{1}H NMR (400 MHz, $CDCl_3$) δ 7.68 (dd, J_1 = 1.2 Hz, J_2 = 4.8 Hz, 1H), 7.64 (m, 2H), 7.60 (dd, J_1 = 1.2 Hz, J_2 = 4.0 Hz, 1H), 7.11-7.15 (m, 6H), 7.05 (m, 2H), 6.94-6.97 (m, 4H), 6.63-6.67 (m, 4H), 3.75 (s, 3H), 3.74 (s, 3H); ^{13}C NMR (100 MHz, $CDCl_3$) δ 187.0, 158.4, 158.3, 143.7, 143.6, 141.8, 138.1, 135.8, 135.7, 135.4, 134.3, 133.7, 132.6, 132.5, 131.4, 131.3, 128.8, 127.8, 127.7, 126.4, 113.2, 113.0, 55.1, 55.0; HRMS (EI) calcd for $[M]^+$: 502.1603, found: 502.1605.

Synthesis of compound 5.

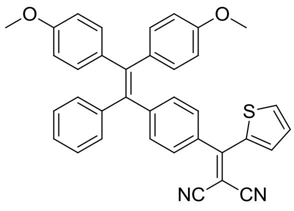 (**5**)

To the solution of compound **4** (0.26 g, 0.52 mmol) and malononitrile (45 mg, 0.68 mmol) in dichloromethane (10 mL) was added titanium tetrachloride (0.20 mL, 1.8 mmol) slowly at 0°C. After the reaction mixture was stirred for 30 min, pyridine (0.15 mL, 1.8 mmol) was injected and stirred for another 30 min. Then the mixture was heated at 40 °C for 4h. After the mixture was cooled down to room temperature, the reaction was quenched by water (10 mL) and the mixture was extracted with dichloromethane. The collected organic layer was washed with brine (20 mL), dried over $MgSO_4$ and concentrated under reduced pressure. The desired residue was purified by column chromatography (hexane/ethyl acetate = 50/1 to 10/1) to give compound **5** as a red solid (230 mg, 81.0% yield). ^{1}H NMR (400 MHz, $CDCl_3$) δ 7.80 (dd, J_1 = 1.2 Hz, J_2 = 5.2 Hz, 1H), 7.73 (dd, J_1 = 1.2 Hz, J_2 = 5.2 Hz, 1H), 7.13-7.22 (m, 8H), 7.06 (m, 2H), 8.91-8.98 (m, 4H), 8.64-8.68 (m, 4H), 3.75 (s, 6H); ^{13}C NMR (100 MHz, $CDCl_3$) δ 164.8, 158.6, 158.4, 148.7, 143.2, 142.4, 138.7, 137.7, 136.1, 135.7, 135.5, 133.5, 132.6, 132.5, 131.5, 131.3, 129.1, 128.8, 127.9, 126.5, 114.5, 113.8, 113.2, 113.0, 55.1, 55.0. MS (EI) calcd for $[M]^+$: 550.1709, found: 550.1708.

Synthesis of compound 6.

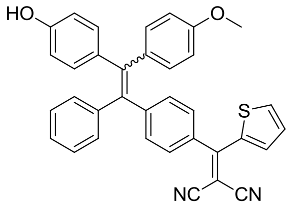 (**6**)

To the solution of compound **5** (170 mg, 0.31 mmol) in dichloromethane (10 mL) was added boron tribromide (1.0 M in dichloromethane, 0.50 mmol) at 0 °C. Then the reaction mixture was stirred at room temperature for 3 h. The reaction was quenched by addition of water (5 mL) under ice-water bath. The organic layer was collected, washed with brine (15 mL), dried over $MgSO_4$ and concentrated under reduced pressure. The desired residue was purified by column chromatography (hexane/ethyl acetate = 20/1 to 5/1) to give compound **6** as a red solid (43 mg, 25.8% yield). ^{1}H NMR (400 MHz, $CDCl_3$) δ 7.79-7.81 (m, 1H), 7.71-7.73 (m, 1H), 7.11-7.22 (m, 8H), 7.06-7.08 (m, 2H), 6.90-6.99 (m, 4H), 6.64-6.68 (m, 2H), 6.57-6.61 (m, 2H), 3.75 (s, 3H); ^{13}C NMR (100 MHz, $CDCl_3$) δ 165.2, 164.9, 158.5, 158.4, 154.9, 154.7, 148.9, 148.7, 143.1, 142.5, 142.3, 138.7, 138.6, 137.7, 137.6, 136.4, 136.2, 136.1, 135.9,

135.5, 135.4, 135.3, 133.5, 133.4, 132.8, 132.7, 132.6, 132.5, 131.5, 131.3, 129.1, 128.9, 128.8, 127.9, 126.5, 114.9, 114.6, 114.4, 113.8, 113.7, 113.2, 113.0, 55.1, 55.0; HRMS (EI) calcd for $[M]^+$: 536.1658, found: 536.1654.

Synthesis of TPETP.

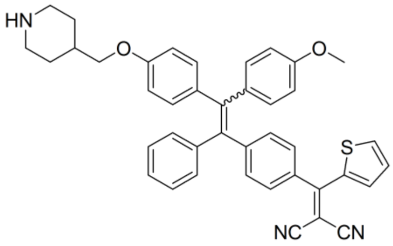

(TPETP)

To the solution of compound **6** (80 mg, 0.15 mmol) in DMF (5 mL) was added tert-butyl 4-(bromomethyl)-piperidine-1-carboxylate (56 mg, 0.20 mmol) and cesium carbonate (62 mg, 0.20 mmol). Then the mixture was stirred at room temperature for 4 h. The mixture was poured into cold water and extracted with ethyl acetate (50 mL). The organic layer was collected, washed by water (50 mL×3) and brine (50 mL×2), dried over $MgSO_4$. The mixture was filtered and the filtrate was concentrated and purified with chromatograpy to give a red solid. The red solid was subsequently dissolved in DCM (5 mL) and TFA (1 mL) and stirred at room temperature for 8 h. Then the solvent was removed and the residue was purified with HPLC to give TPETP as a red solid (20 mg, 21.0% yield). ^{1}H NMR (400 MHz, DMSO-d_6) δ 8.61 (m, 1H), 8.27 (dd, J_1 = 1.2 Hz, J_2 = 5.2 Hz, 1H), 7.66 (dd, J_1 = 1.2 Hz, J_2 = 5.2 Hz, 1H), 7.32-7.38 (m, 3H), 7.09-7.19 (m, 5H), 7.04 (m, 2H), 6.92 (m, 2H), 6.85 (m, 2H), 6.68-6.72 (m, 4H), 3.79 (d, J = 6.4 Hz, 2H), 3.67 (s, 3H), 3.31 (m, 2H), 2.91 (m, 2H), 2.00 (m, 1H), 1.90 (m, 2H), 1.45 (m, 2H); MS (ESI) calcd for $[M+H]^+$:634.25, found: 634.20. HRMS (ESI) calcd for $[M+H]^+$:634.2523, found: 634.2525.

Synthesis of TPETP-AA-Rho-N$_3$.

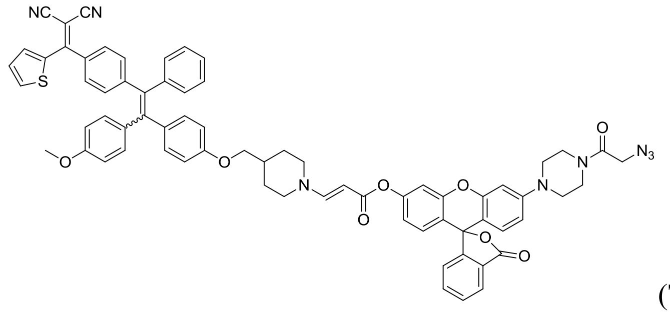

(**TPETP-AA-Rho-N$_3$**)

To the solution of TPETP (8.0 mg, 0.01 mmol) in THF (5 mL) was added TEA (2.0 mg, 0.02 mmol) and Rho (5.4 mg, 0.01 mmol). The reaction was stirred at room temperature for 1 h. Then the mixture was run through

chromatography (hexane: ethyl acetate = 1:1) to give TPETP-AA-Rho-N_3 as a yellow solid (5.0 mg, 37.3% yield). HRMS (ESI) calcd for $[M+Na]^+$:1191.3834, found: 1191.3836.

"Click" Synthesis of TPETP-AA-Rho-cRGD. Alkyne-functionalized peptide cRGD (3.8 mg, 14 μmol) and TPETP-AA-Rho-N_3 (5.2 mg, 4.4 μmol) were dissolved in a mixture of dimethyl sulfoxide and water (v/v = 5/1, 0.6 mL). The "click" reaction was initiated by sequential addition of $CuSO_4$ (0.6 mg, 4.4 μmol) and sodium ascorbate (1.6 mg, 8 μmol). The reaction was continued with stirring at room temperature in dark for another 24 h. The final product was purified by preparative HPLC and lyophilized under vacuum to yield the probe (2.9 mg, 38% yield) as a yellow powder. HPLC (λ = 214 nm): purity 98.4%. ESI-MS: *m/z* $[M+2H]^{2+}$ calc. 870.825, found 870.832.

Singlet Oxygen Detection in Solution. The singlet oxygen generation was studied using ABDA as an indicator as the absorbance of ABDA decreases upon reaction with singlet oxygen.[2] For singlet oxygen detection, the ABDA (200 μM) was mixed with the PS (10 μM) in DMSO/PBS (v/v = 1/199) and exposed to visible light (λ = 400–700 nm) irradiation. The decomposition of ABDA was monitored by the absorbance decrease at 400 nm.

Singlet Oxygen Quantum Yield Measurements.[3] The singlet oxygen generated from the PSs upon visible light irradiation (400–700 nm) was studied by using ABDA as an indicator. The absorbance decrease of ABDA at 400 nm was recorded for different durations of light irradiation to obtain the decay rate of the photosensitizing process. Using Rose Bengal (RB) as a reference, the singlet oxygen quantum yield of TPETP (Φ_{PS}) was calculated according to the following formula:

$$\Phi_{\mathrm{PS}} = \Phi_{\mathrm{RB}} \frac{K_{PS} * A_{RB}}{K_{RB} * A_{PS}}$$

Where K_{PS} and K_{RB} are the decomposition rate constants of ABDA by TPETP and RB. A_{PS} and A_{RB} represent the light absorbed by TPETP and RB, which are determined by integration of the absorption bands in the wavelength range of 400–700 nm. Φ_{RB} is the singlet oxygen quantum yield of RB, which is 0.75 in water.

Selectivety of the Probe. DMSO stock solution of the probe (1 mM) was diluted into a mixture solvent of DMSO and PBS (v/v = 1/199). Then the probe (10 μM) was incubated with different kinds of ROS (10 μM) at room temperature and the fluorescence change of Rho was studied. H_2O_2, $ONOO^-$, and $O_2^{\bullet-}$ were prepared by directly diluting commercially available H_2O_2, NaONOO and KO_2 respectively. $^{\bullet}OH$ was

generated from Fenton reaction between H_2O_2 and $Fe(ClO_4)_2$. $^1O_2$ was produced from the H_2O_2/molybdate ions (Na_2MoO_4) system.

Cell Culture. U87-MG human glioblastoma cancer cells, MDA-MB-231 and MCF-7 human breast cancer cells, HepG2 human hepatoma cancer cells, 293T and NIH/3T3 normal cells were provided by American Type Culture Collection (ATCC). The cells were cultured in DMEM medium containing 100 μg mL^{-1} streptomycin, 10% heat-inactivated FBS, 100 U mL^{-1} penicillin, and maintained in a humidified incubator with 5% CO_2 at 37 °C.

Intracellular ROS Detection. The intracellular ROS generation was detected by using 2′,7′-dichlorofluorescin diacetate (DCF-DA) as an indicator and studied by confocal images. MDA-MB-231 and U87-MG cells in 8-well chambers (Thermo Scientific) were firstly incubated with TPETP (5 μM) for 4 h in the dark, then the cells were rinsed with PBS for 3 times and stained with 2 μM of DCF-DA. After 10 min incubation, the cells were washed with 1× PBS and exposed to visible light (λ = 400–700 nm) irradiation (30 s, 0.10 W cm^{-2}). After irradiation, the cells were washed with 1× PBS and studied by confocal microscope (CLSM, Zeiss LSM 410, Jena, Germany). For DCF detection, the excitation wavelength was 488 nm, and the emission filter was 505−525 nm.

Confocal Imaging. The cells in 8-well chambers were prepared according to the above description and incubated with the probe for 4 h. After incubation with the probe, the cells were washed with PBS and the cell nuclei were live stained with Hoechst 33342, following standard protocols of the manufacturer (Life Technologies) and imaged immediately by confocal microscope. To confirm the cellular uptake *via* $\alpha_v\beta_3$ integrin mediated endocytosis, a competition assay was performed by adding an excessive of cRGD (100 μM) into the culture media. For Hoechst 33342 detection, the excitation wavelength was 405 nm, and the emission filter was 430−470 nm; for Rho detection, the excitation wavelength was 488 nm, and the emission filter was 505−525 nm; for TPETP detection, the excitation was 405 nm, and the emission was collected above 650 nm.

Flow Cytometry Study. MDA-MB-231 and U87-MG cells in 24-well plate (Costar, IL, USA) were precultured overnight and incubated with the probe for the designated time and exposed to visible light (λ = 400–700 nm) irradiation. After incubation, the cells were treated with trypsin, washed with medium twice and subjected to flow cytometry analysis using Cyan-LX (DakoCytomation). The cells without any treatment were used as control. The mean fluorescence was determined by counting 10,000 events.

Cytotoxicity Studies. MTT assays were used to assess the cell viability of MDA-MB-231, U87-MG, MCF-7, HepG2, NIH/3T3 and 293T cells after incubation with the probe and exposed with white light irradiation. The cells

in 96-well plates (Costar, IL, USA) were first incubated with the probe for designated time in the dark. Then the cells were washed with PBS twice and exposed to white light irradiation at a power density of 0.10 W cm^{-2}. The cells were further incubated in fresh medium for 24 h and washed with PBS. Then MTT in PBS solution (100 μL, 0.5 mg mL^{-1}) was added into each well. After incubation for 3 h, the supernatant was discarded and the precipitate was dissolved in DMSO (100 μL) with gentle shaking. The absorbance of MTT at 570 nm was monitored by the microplate reader (Genios Tecan). The cells without any treatment were used as control.

References:

(1) (a) F. Hu, Y. Y. Huang, G. X. Zhang, R. Zhao, H. Yang, D. Q. Zhang, *Anal. Chem.* **2014**, *86*, 7987; (b) Y. Yuan, C. J. Zhang, M. Gao, R. Zhang, B. Z. Tang, B. Liu, *Angew. Chem. Int. Ed.* **2015,** 54, 1780.

(2) Gomes, A.; Fernandes, E.; Lima, J. L. F. C. *J. Biochem. Bioph. Meth.* **2005**, *65*, 45.

(3) J. C. Ge, M. H Lan, B. J. Zhou, W. M. Liu, L. Guo, H. Wang, Q. Y. Jia, G. L. Niu, X. Huang, H. Y. Zhou, X. M. Meng, P. F. Wang, C. S. Lee, W. J. Zhang, X. D. Han, *Nat. Commun.* **2014**, 5, 4596.

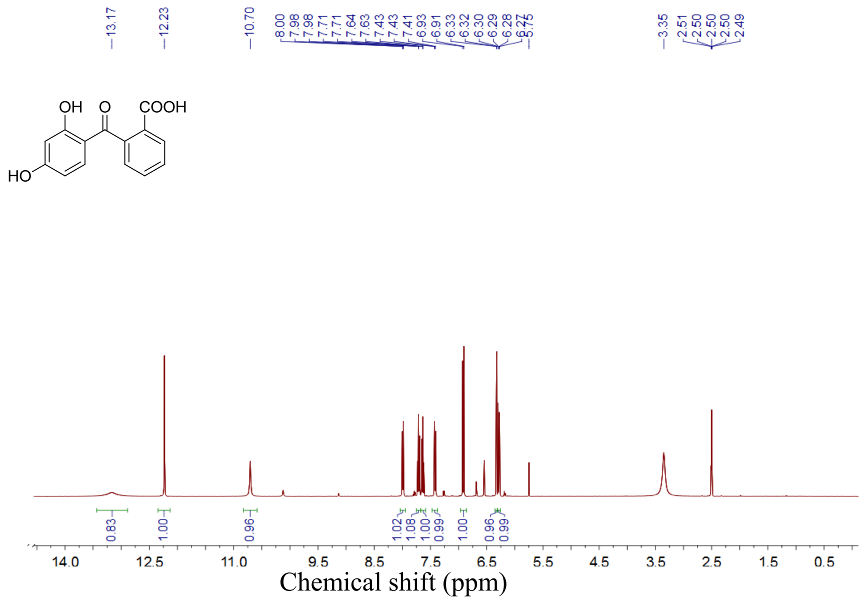

Figure S1. ^{1}H NMR spectrum of compound **1**.

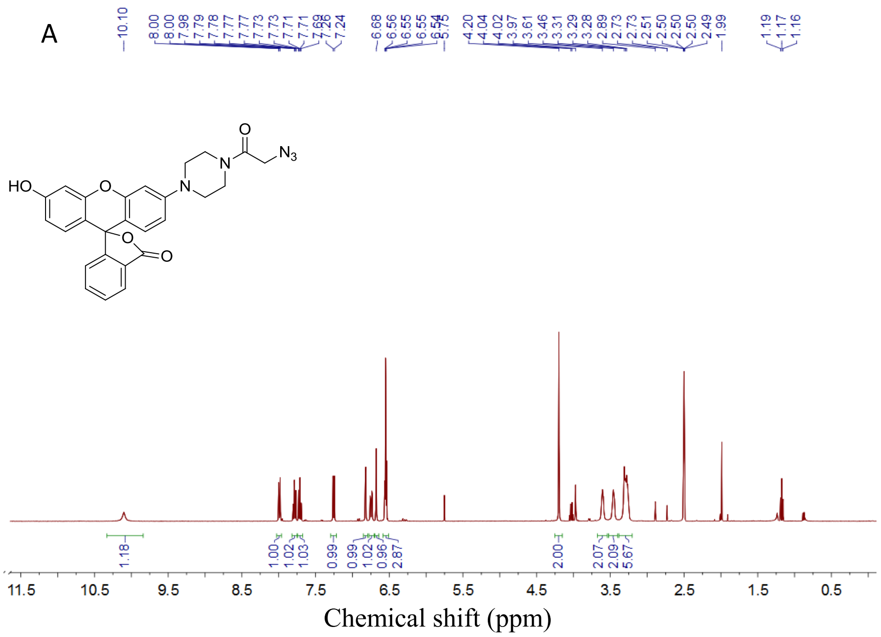

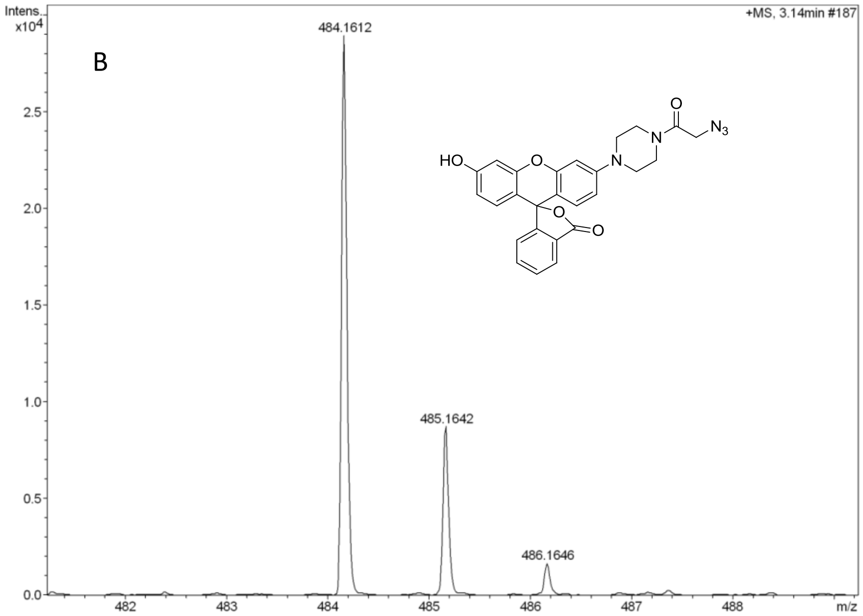

Figure S2. (A) ^{1}H NMR (A) and high resolution mass spectra (B) of compound **2**.

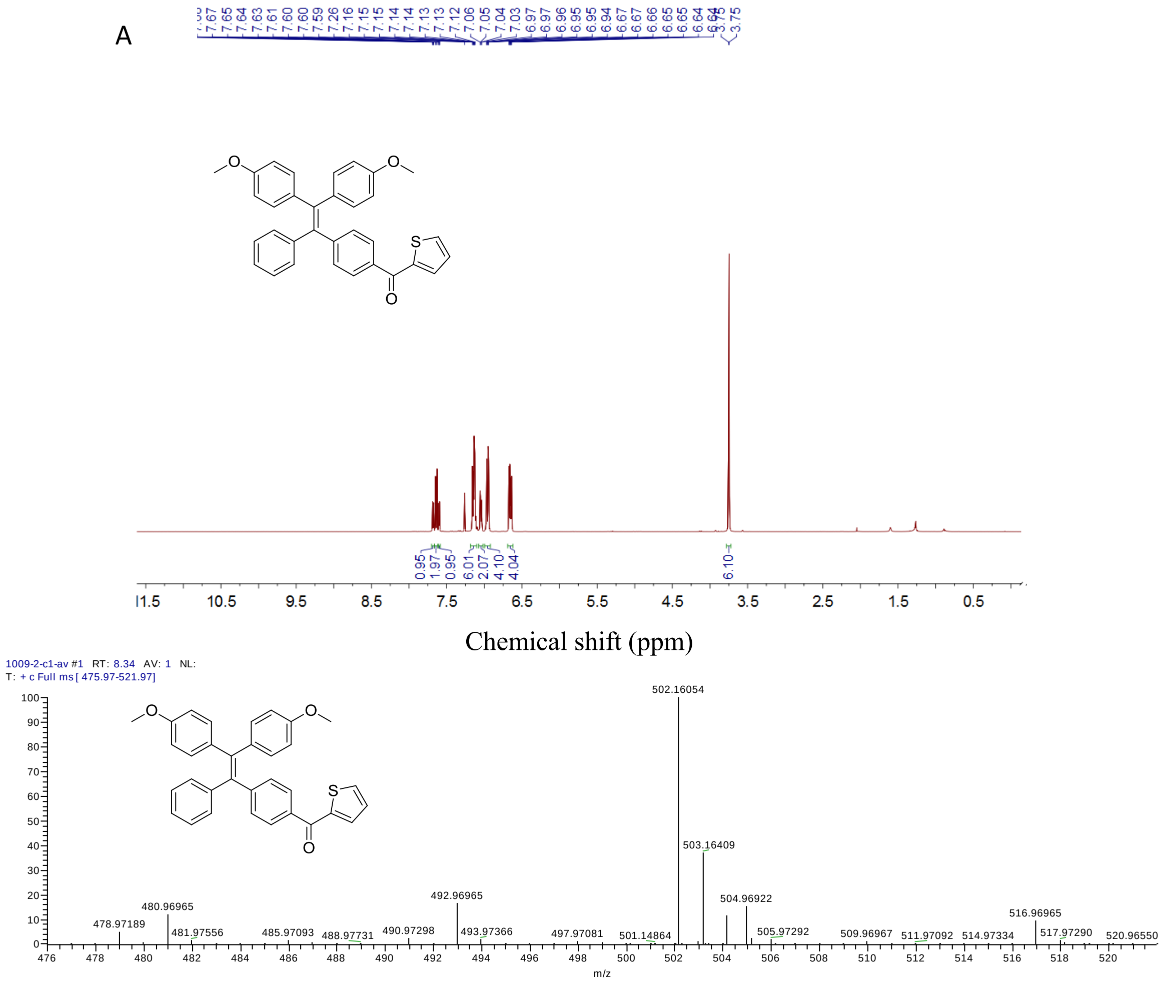

Figure S3. ^{1}H NMR (A) and high resolution mass spectra (B) of compound **4**.

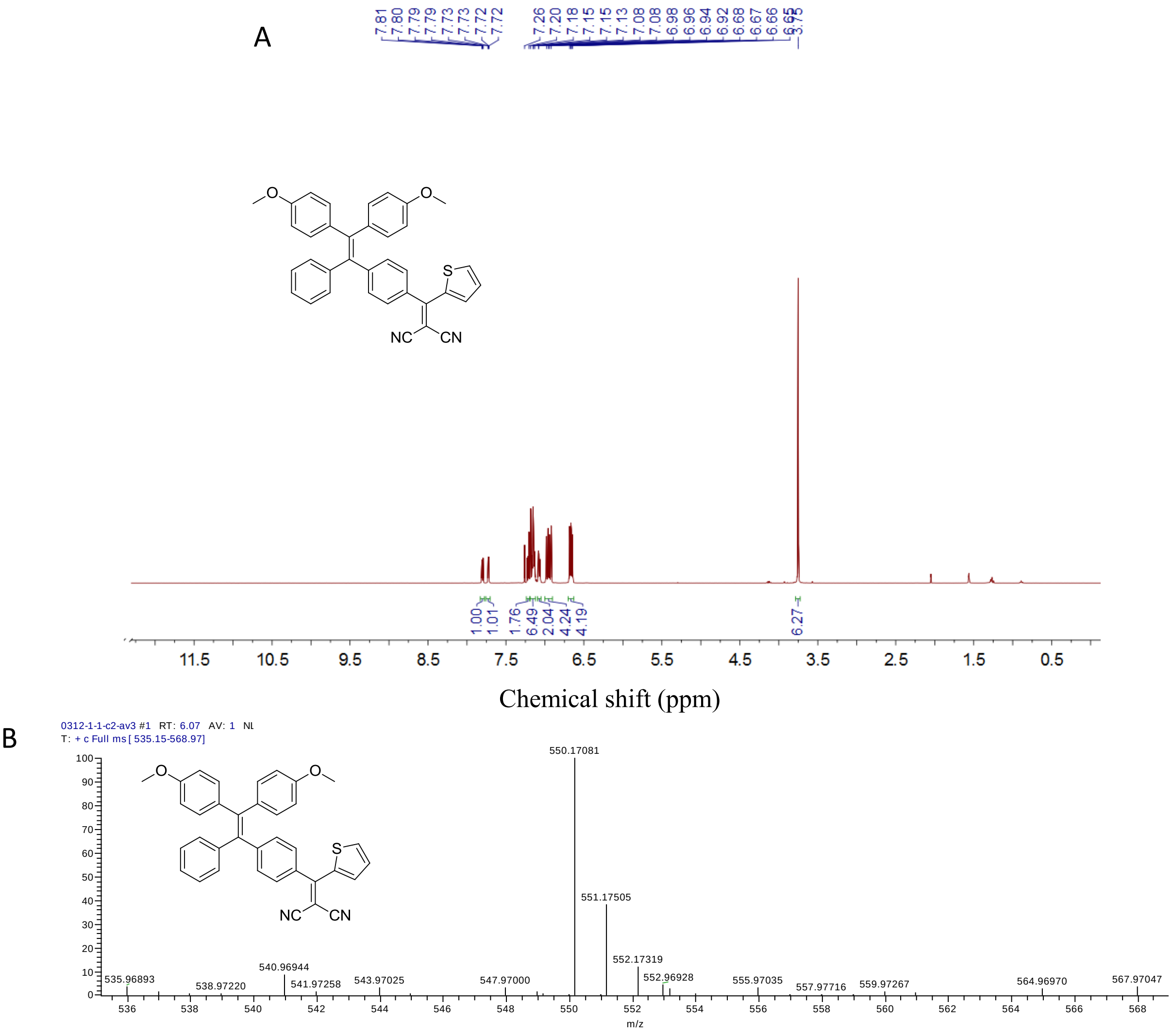

Figure S4. ^{1}H NMR (A) and high resolution mass spectra (B) of compound **5**.

A

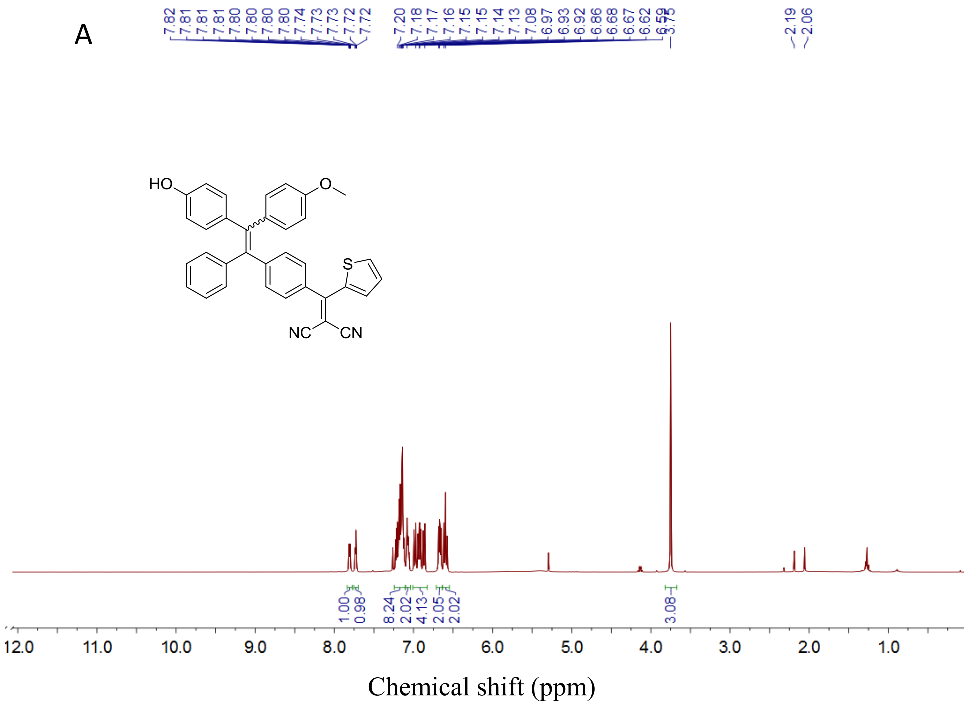

B

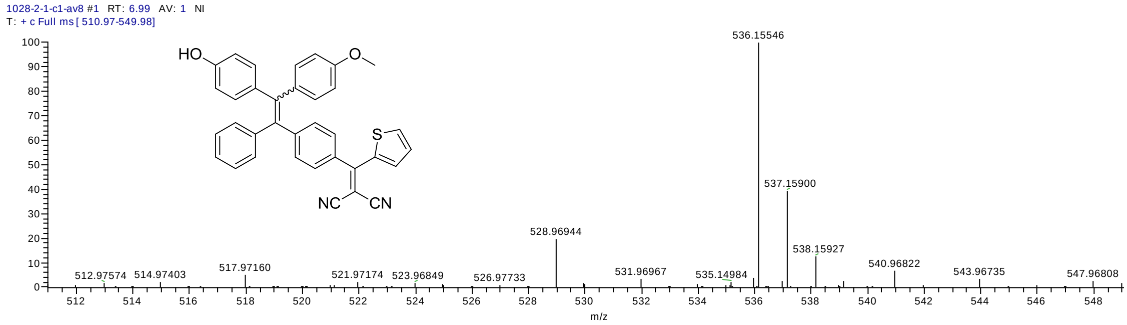

Figure S5. ^{1}H NMR (A) and high resolution mass spectra (B) of compound **6**.

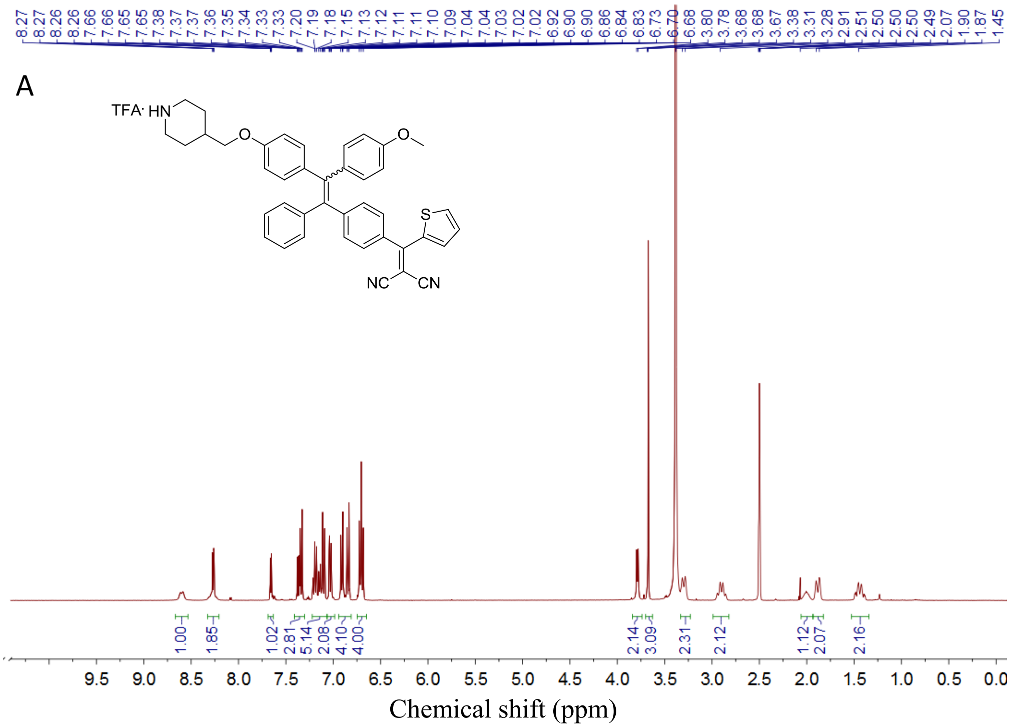

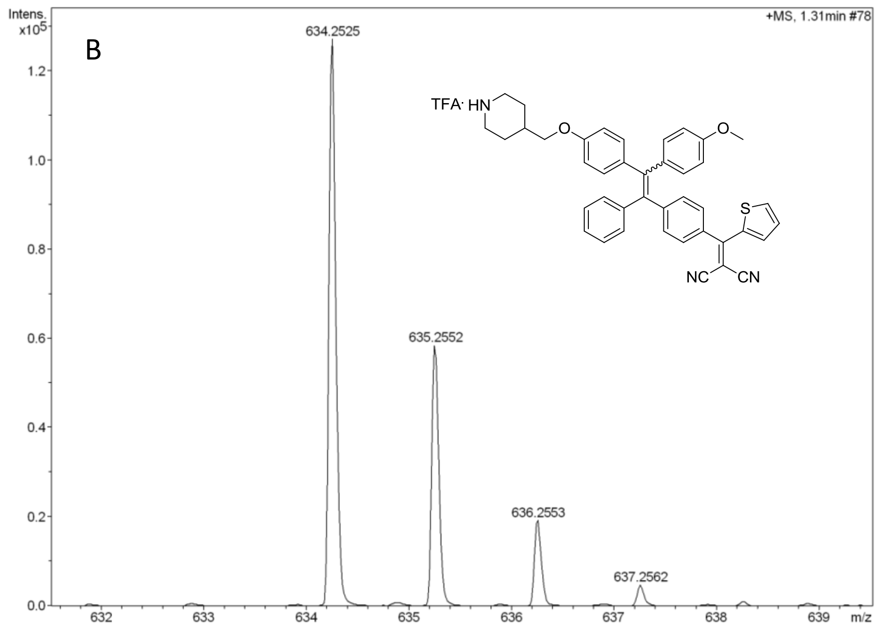

Figure S6. ^{1}H NMR (A) and high resolution mass spectra (B) of TPETP.

A

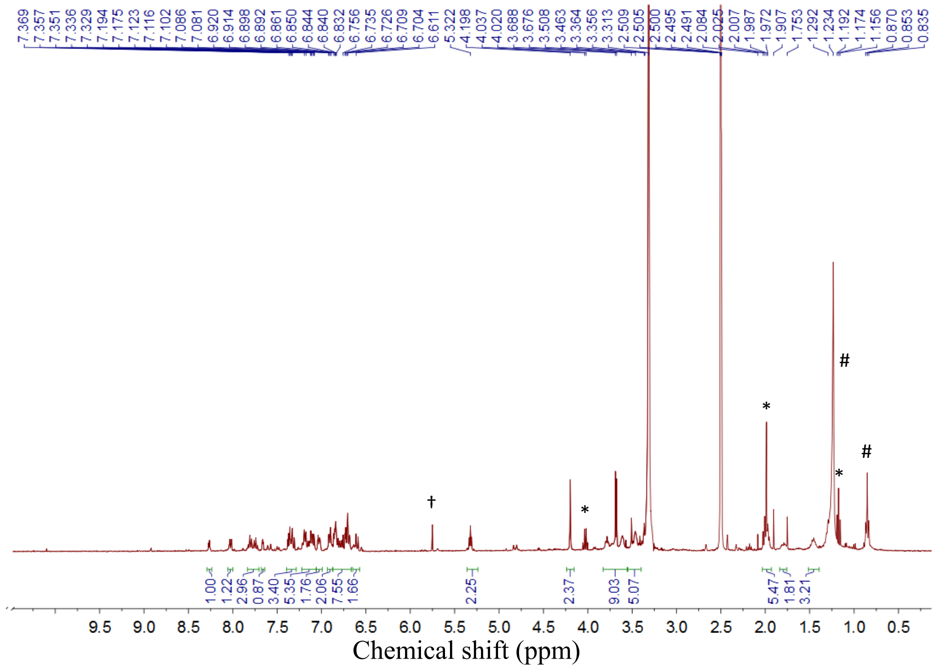

B

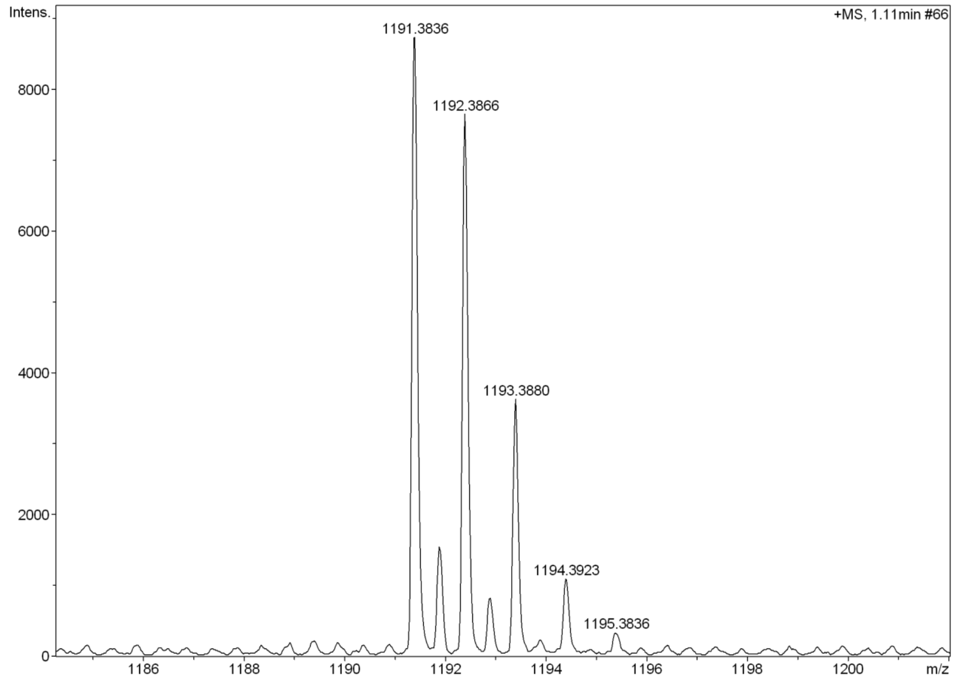

Figure S7. ^{1}H NMR (A) and high resolution mass spectra (B) of TPETP-AA-Rho-N_3. * are the protons from ethyl acetate; † are the protons from dichloromethane; # are the protons from hexane.

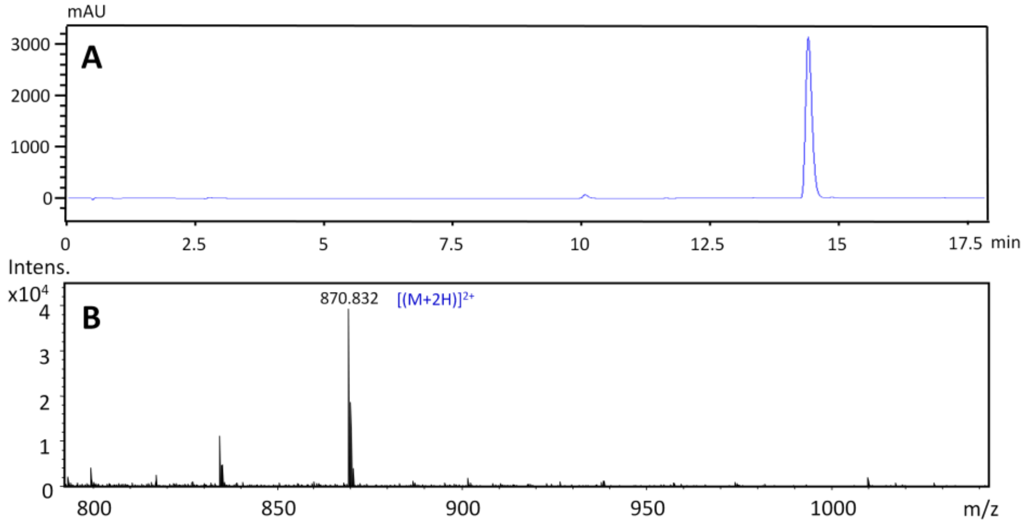

Figure S8. HPLC (A) and Mass (B) spectra of the probe TPETP-AA-Rho-cRGD.

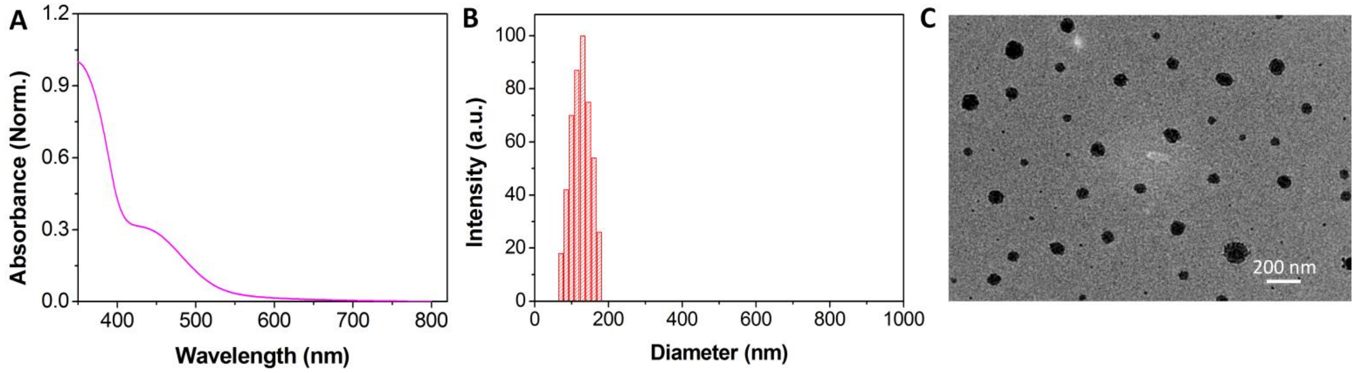

Figure S9. (A) UV-vis absorption spectrum of the TPETP in DMSO/H_2O (v/v = 1/199); Laser light scattering (LLS) data (B) and TEM image (C) of TPETP in DMSO/H_2O (v/v = 1/199).

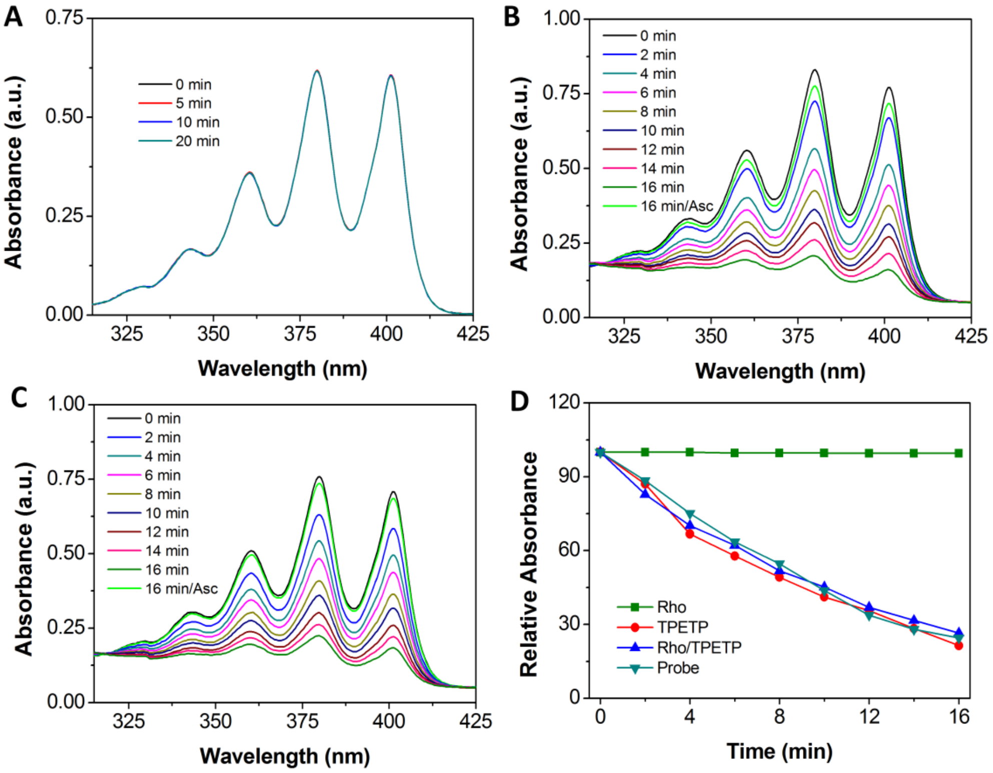

Figure S10. UV-vis absorption spectra of singlet oxygen indicator 9,10-anthracenediyl-bis(methylene)dimalonic acid (ABDA) mixed with (A) Rho, (B) TPETP or (C) Rho and TPETP mixture. Asc stands for singlet oxygen scavenger ascorbic acid. (D) Plots of the changes in absorbance of ABDA at 400 nm *vs* irradiation time after light irradiation (0.10 W cm^{-2}).

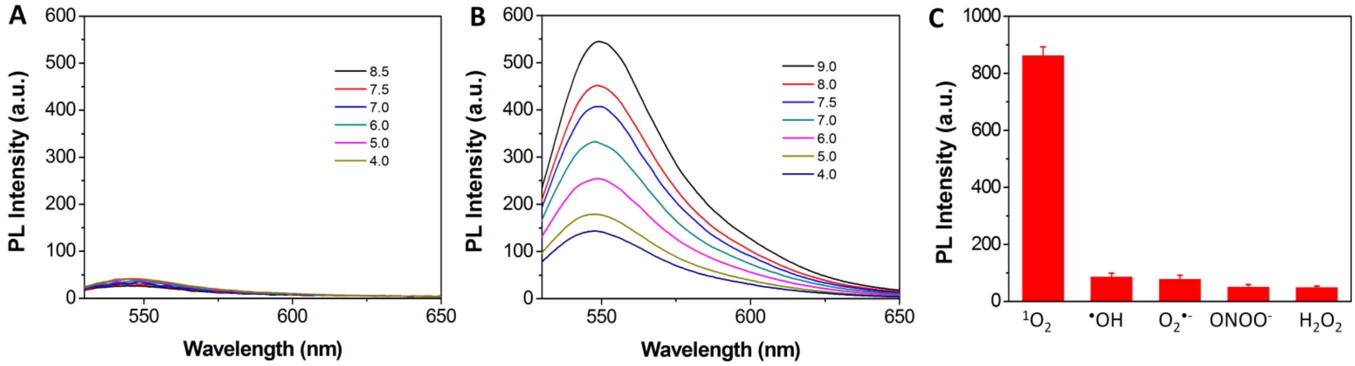

Figure S11. PL intensity change of the probe (10 μM) before (A) and after (B) light irradiation at different pH. The excitation wavelength was 510 nm. (C) Fluorescence intensity of Rho after the probe (10 μM) was incubated with different kinds of ROS in N_2-purged PBS (pH 7.4).

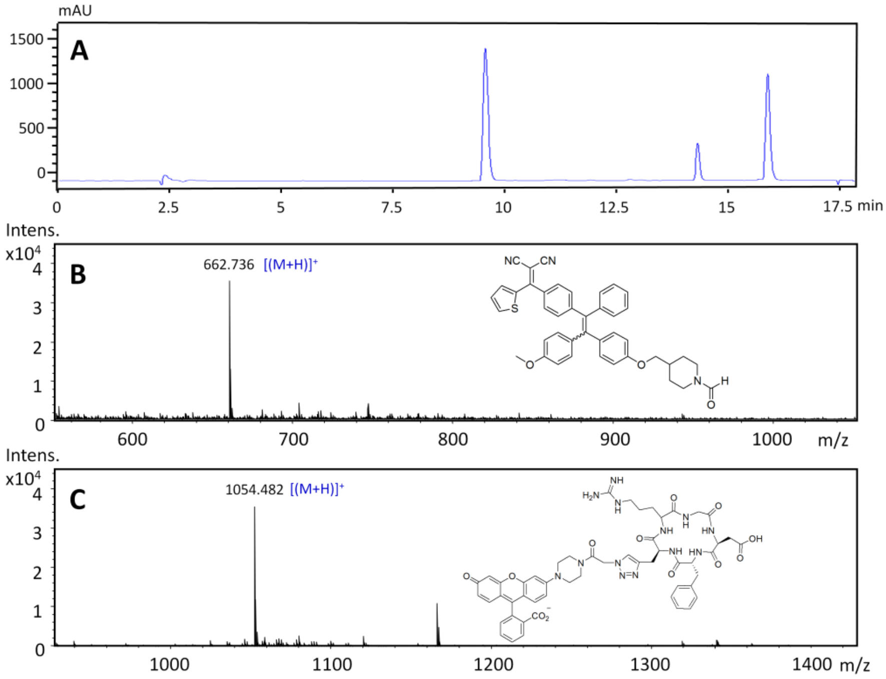

Figure S12. (A) HPLC analysis of the photo-products of TPETP-AA-Rho-cRGD after exposure to light at the power density of 0.1 W cm^{-2} for 5 min. Peaks in the chromatograms were detected by monitoring the UV/Vis absorbance at 405 nm. The corresponding mass spectra of the peak at 9.6 min (B) and 15.9 min (C) determined by ESI-MS spectrometry.

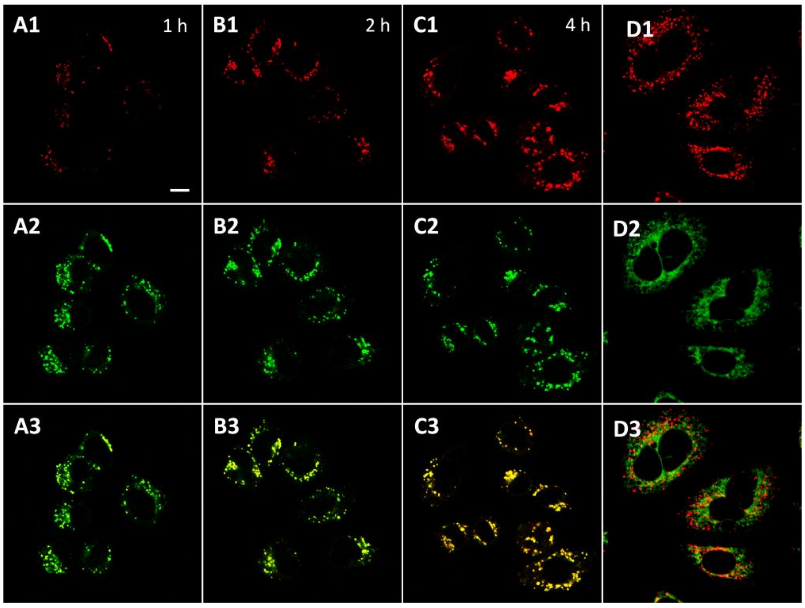

Figure S13. CLSM images of MDA-MB-231 cells upon incubation with the probe (A1-D1) for different time durations and co-stained with LysoTracker Red (A2-C2) or MitoTracker Red (D2). (A1-D1) The red fluorescence is from the TPETP (E_x: 405 nm; E_m: > 650 nm); (A2-D2) the green fluorescence is from LysoTracker or MitoTracker (E_x: 543 nm, E_m: 610−640 nm). A3-D3 are the overlay images of A1-D1 and A2-D2, respectively. All the images share the same scale bar of 10 μm.

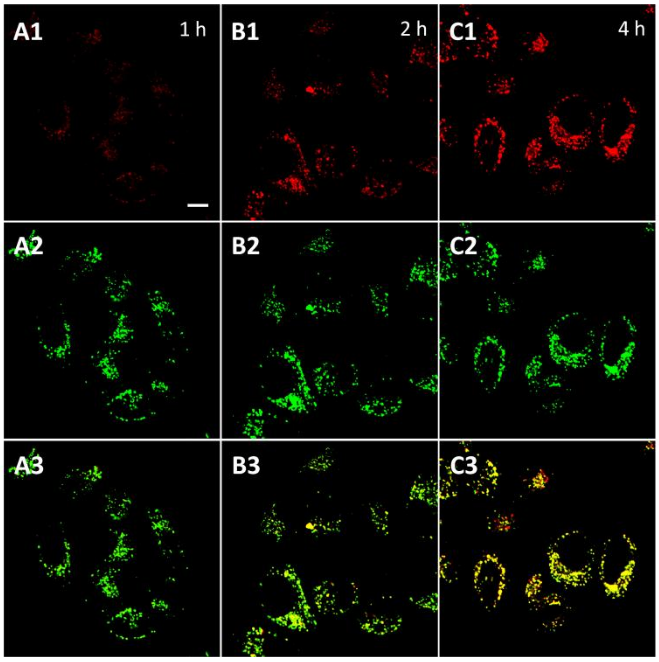

Figure S14. CLSM images of U87-MG cells upon incubation with the probe (A1-C1) for different time durations and co-stained with LysoTracker Red (A2-C2). (A1-C1) The red fluorescence is from TPETP (E_x: 405 nm; E_m: > 650 nm); (A2-C2) the green fluorescence is from LysoTracker or MitoTracker (E_x: 543 nm, E_m: 610−640 nm). A3-C3 are the overlay images of A1-C1 and A2-C2, respectively. All the images share the same scale bar of 10 μm. Due to the low absorbance of TPETP at 543 nm, its spectral overlap with LysoTracker or MitoTracker is negligible.

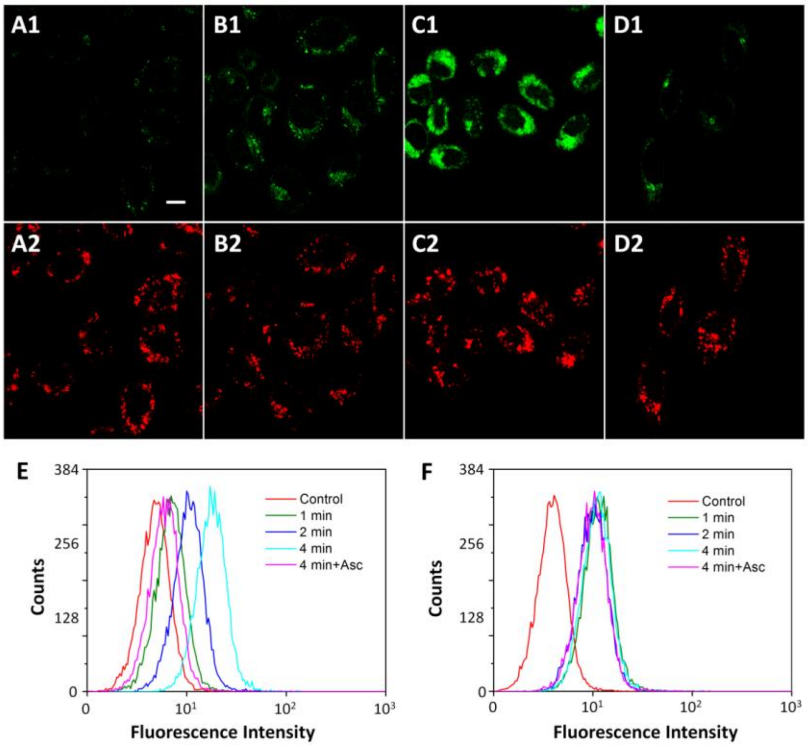

Figure S15. (A-D) CLSM images of U87-MG cells upon incubation with the probe for 4 h and exposed with light irradiation for (A) 0 min, (B) 1 min, (C) 4 min and (D) 4 min in the presence of Asc at a power intensity of 0.10 W cm^{-2} in fresh medium. The red fluorescence is from the TPETP (E_x: 405 nm; E_m: > 650 nm); the green fluorescence is from Rho (E_x: 488 nm, E_m: 505−525 nm). A3-D3 are the overlay of the images A1-D1 and A2-D2, respectively. All the images share the same scale bar of 10 μm. (E-F) Flow cytometric analysis of Rho (E) and TPETP (F) fluorescence after light irradiation.

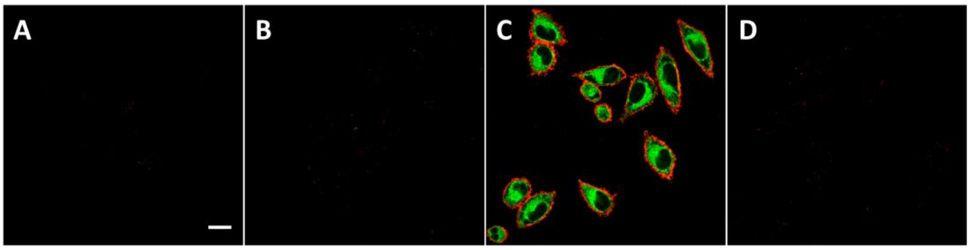

Figure S16. Cell apoptosis imaging of U87-MG cells stained with Annexin V-Cy5 (A), incubated with the probe only (B), Annexin V-Cy5/probe with light irradiation (C) or in the presence of Asc (D). The power density was 0.10 W cm^{-2}. The green fluorescence is from the Rho (E_x: 488 nm; E_m: 505-525 nm); the red fluorescence is from Cy5 (E_x: 633 nm, E_m: > 650 nm). All the images share the same scale bar of 20 μm.

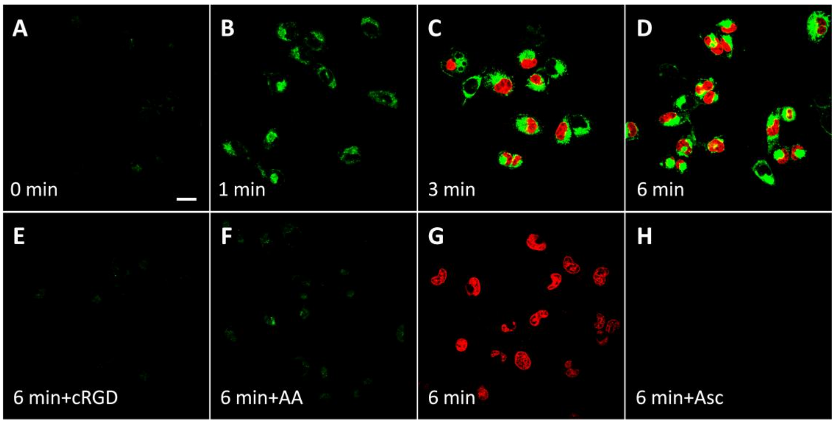

Figure S17. CLSM images of the cell necrosis induced by the probe (A-F) or TPETP (G, H) incubated U87-MG cell with light irradiation at a power density of 0.10 W cm^{-2} for different time durations and further incubated for 4 h in fresh medium before staining with propidium iodide (PI). The green fluorescence is from the Rho (E_x: 488 nm; E_m: 505-525 nm); the red fluorescence is from PI (E_x: 543 nm, E_m: 610−640 nm). All the images share the same scale bar of 10 μm. Due to the low absorbance of TPETP at 543 nm, the spectral overlap with PI is negligible.

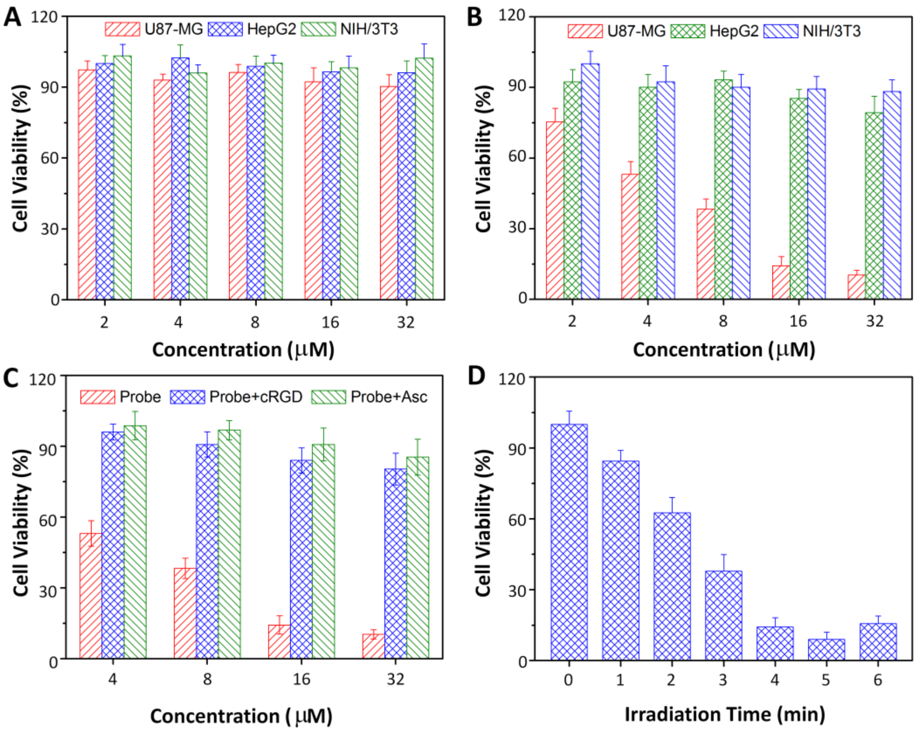

Figure S18. Cytotoxicity of U87-MG, HepG2 and NIH/3T3 cells upon incubation with the probe (A) in dark conditions or (B) under light irradiation (4 min, 0.10 W cm^{-2}). (C) Cytotoxicity of the probe to U87-MG cells upon pretreatment with 100 μM of cRGD or Asc, followed by light irradiation (4 min, 0.10 W cm^{-2}). (D) Cytotoxicity of the probe to U87-MG cells with light irradiation for different time durations.

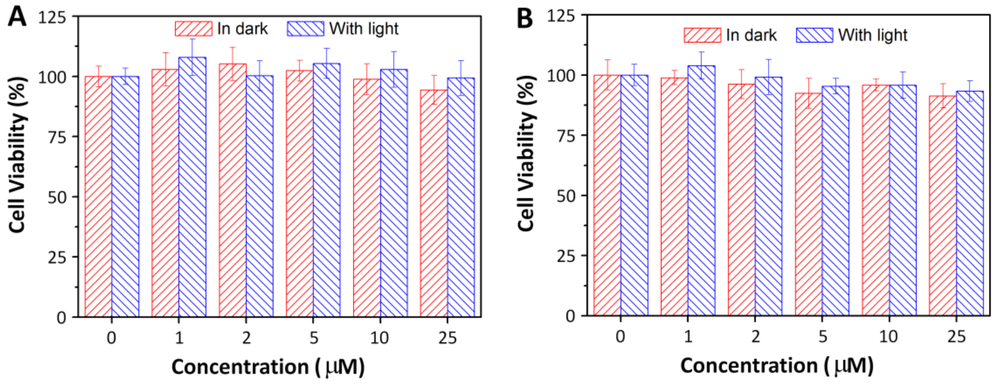

Figure S19. Cell viability of MDA-MB-231 (A) and U87-MG (B) cells upon treatment with Rho at different concentrations in dark or with light irradiation at a power density of 0.10 W cm^{-2} for 4 min and further incubation in fresh medium for 24 h.
